# Trace amine-associated receptor 1 regulation of Kv1.4 channels in trigeminal ganglion neurons contributes to nociceptive behaviors

**DOI:** 10.1186/s10194-023-01582-5

**Published:** 2023-05-08

**Authors:** Yuan Zhang, Hua Wang, Yufang Sun, Zitong Huang, Yu Tao, Yiru Wang, Xinghong Jiang, Jin Tao

**Affiliations:** 1 Department of Geriatrics & Clinical Research Center of Neurological Disease, The Second Affiliated Hospital of Soochow University, 1055 San-Xiang Road, Suzhou, 215004 P.R. China; 2 Department of Physiology and Neurobiology & Centre for Ion Channelopathy, Medical College of Soochow University, 199 Ren-Ai Road, Suzhou, 215123 P.R. China; 3 Jiangsu Key Laboratory of Neuropsychiatric Diseases, Soochow University, Suzhou, 215123 P.R. China; 4 Department of Endocrinology, Shanghai East Hospital, Tongji University School of Medicine, Shanghai, 200120 P.R. China

**Keywords:** Trace amine-associated receptor 1, Potassium channels, Migraine, Trigeminal ganglion neurons, Protein kinase C

## Abstract

**Background:**

Trace amines, such as tyramine, are endogenous amino acid metabolites that have been hypothesized to promote headache. However, the underlying cellular and molecular mechanisms remain unknown.

**Methods:**

Using patch-clamp recording, immunostaining, molecular biological approaches and behaviour tests, we elucidated a critically functional role of tyramine in regulating membrane excitability and pain sensitivity by manipulating Kv1.4 channels in trigeminal ganglion (TG) neurons.

**Results:**

Application of tyramine to TG neurons decreased the A-type K^+^ current (*I*_A_) in a manner dependent on trace amine-associated receptor 1 (TAAR1). Either siRNA knockdown of Gαo or chemical inhibition of βγ subunit (G_βγ_) signaling abrogated the response to tyramine. Antagonism of protein kinase C (PKC) prevented the tyramine-induced *I*_A_ response, while inhibition of conventional PKC isoforms or protein kinase A elicited no such effect. Tyramine increased the membrane abundance of PKC_θ_ in TG neurons, and either pharmacological or genetic inhibition of PKC_θ_ blocked the TAAR1-mediated *I*_A_ decrease. Furthermore, PKC_θ_-dependent *I*_A_ suppression was mediated by Kv1.4 channels. Knockdown of Kv1.4 abrogated the TAAR1-induced *I*_A_ decrease, neuronal hyperexcitability, and pain hypersensitivity. In a mouse model of migraine induced by electrical stimulation of the dura mater surrounding the superior sagittal sinus, blockade of TAAR1 signaling attenuated mechanical allodynia; this effect was occluded by lentiviral overexpression of Kv1.4 in TG neurons.

**Conclusion:**

These results suggest that tyramine induces Kv1.4-mediated *I*_A_ suppression through stimulation of TAAR1 coupled to the G_βγ_-dependent PKC_θ_ signaling cascade, thereby enhancing TG neuronal excitability and mechanical pain sensitivity. Insight into TAAR1 signaling in sensory neurons provides attractive targets for the treatment of headache disorders such as migraine.

**Supplementary Information:**

The online version contains supplementary material available at 10.1186/s10194-023-01582-5.

## Background

Tyramine, a trace amine derived from the metabolism of amino acids, is naturally found in foods and plants and has been endogenously identified in the mammalian brain and peripheral nervous tissues [1]. Although several trace amine-associated receptors (TAARs) have been identified, the primary endogenous targets of tyramine are TAAR1 and, to a lesser extent, TAAR4 [2]. Acting through its receptors, tyramine mediates a variety of important biological effects, such as promoting intracranial hemorrhages, blurry vision, and myocardial injury [3, 4]. Recently, emerging evidence has also revealed the functional role of tyramine/TAARs in nociception [5]. For instance, intracerebral administration of the TAAR1 agonist 3-iodothyronamine decreased the threshold to painful stimuli in mice [6, 7]. Ingestion of foods containing high levels of tyrosine by patients taking inhibitors of monoamine oxidase, the most notable tyramine-degrading enzyme, produces headaches and chest pain [5]. Further findings supporting this hypothesis come from clinical evidence that patients with headache have higher blood levels of tyramine [8, 9]. More interestingly, patients with chronic idiopathic temporomandibular joint and orofacial pain and existing tyramine conjugation deficits excrete significantly lower amounts of tyramine sulfate than controls [10]. However, whether and how tyramine participates in peripheral nociceptive responses remain unknown.

Changes in the neuronal excitability of peripheral sensory neurons may influence nociceptive behaviors [11]. Voltage-gated potassium channels (Kvs) are pivotal regulators of membrane excitability [12]. Kv currents in nociceptive sensory neurons have been classified as the transient outward (A-type) current (*I*_A_) or the delayed rectifier current (*I*_DR_) [13–15]. The *I*_A_ is sensitive to 4-aminopyridine (4-AP), with the characteristics of rapid activation and inactivation, and repolarizes neurons after action potentials [16–18]. Five mammalian Kv α-subunits, Kv4.1, Kv4.2, Kv4.3, Kv1.4, and Kv3.4, expressed in sensory neurons mediate *I*_A_ [15, 19]. These channels play critical roles in contributing to action potential repolarization and have been widely implicated in pain plasticity [15, 16]. For instance, downregulation of Kv4.1 induced by peripheral nerve injury was found to lead to sensory neuronal hyperexcitability, thereby resulting in increased nociceptive responsiveness to mechanical stimuli [20]. Moreover, genetic and functional analyses have identified pivotal roles of Kv3.4 and Kv1.4 channels in amplifying peripheral nociceptive signals and promoting central sensitization [14–16, 21]. Therefore, manipulation of *I*_A_ channels might regulate sensory neuronal excitability, which is considered useful in producing pain relief.

The current study examined the role of TAAR1 in modulating Kv1.4-mediated *I*_A_ and elucidated the underlying mechanisms that elicit nociceptive responses to tyramine. We reported that stimulation of TAAR1 by tyramine triggers the release of the G_o_ protein βγ subunits and subsequently activates downstream PKC_θ_ signaling. TAAR1-Kv1.4-mediated *I*_A_ suppression results in TG neuronal hyperexcitability, which contributes to pain hypersensitivity in mice.

## Materials and methods

### Animal model and behavioral tests

Experimental procedures were carried out in accordance with National Institutes of Health (NIH) guidelines and were approved by the Institutional Animal Care and Use Committee of Soochow University. Animals were housed on a standard 12/12-h light–dark cycle in a temperature- and humidity-controlled room with food and water provided *ad libitum*. Every effort was made to minimize both the number of animals used and animal suffering. The investigators were not blinded to group allocations in experiments other than behavioral experiments. Nociceptive behaviors of migraine were induced by the electrical stimulation of the dura mater surrounding the superior sagittal sinus [22]. The surgical procedures were performed according to previous studies [19, 23] with some modifications. Briefly, mice were anaesthetized and fixed on a stereotaxic apparatus. An incision was made sagittally along the midline, and the parietal bone was clearly exposed. Two cranial windows with 0.5 mm diameters (located 2.4 mm anterior and 3.6 mm posterior to Bregma along the midline suture) were prepared by drilling into the skull to expose the dura mater surrounding the superior sagittal sinus. A pair of stimulating electrodes (0.3 mm in diameter) was placed on the dural surface and fixed to the skull using zinc phosphate cement. After surgery, all animals were allowed to recover for 1 week. Animals received the electrical stimuli with a frequency of 2 Hz and a duration of 250 µs at 0.4 mA, 1 h per day for 5 consecutive days. The animals in the sham groups were treated similarly but did not undergo electrical stimulation. Orofacial behavioral tests were determined with an ascending series of *von* Frey filaments (0.02 to 2.56 g, Ugo Basile) applied to the periorbital region as described previously [24–28]. Each von Frey stimulation was applied three times in each series of trials with an interval of at least 1 min, each lasting 2 − 3 s. The stimulation is expected to result in behavior responses of head withdrawal or escape according to the method initially proposed by Vos et al. [29].

### Drug microinjection into the TG

As described in our previous studies [30–32], drugs/reagents were applied through a percutaneous approach by injecting the TG (Intra-TG injection) with a 30-gauge needle inserted through the infraorbital foramen, infraorbital canal and foramen rotundum. The tip of the needle terminated at the medial part of the TG, and drugs/reagents were slowly delivered in a volume of 3 µl over a 5 min period. 5’-Cholesteryl-modified and 2’-O-methyl-modified small interfering RNA (siRNA) for TAAR1 (TAAR1-siRNA, 5’-GCTTCCCTGTACAGCTTAATG-3’), PKC_θ_-siRNA (5’-GCGACTTAATGTACCACATCC-3’), Gαo-siRNA (5’-CGAATAATAT CCAGGTGGTAT-3’) Gαi-siRNA (5’-GAATCTGGAAAGAGTACCATT-3’), Kv1.4-siRNA (5’-CCTACCTTCT AATTTGCTCAA-3’) or the corresponding scrambled control siRNAs (RiboBio Biological Technology), labeled with Cy3, were dissolved in RNase-free water. Neuron promoter-specific (human synapsin 1 gene promoter, hSyn) combinatorial lentiviral vectors, including lenti-hSyn-Kv1.4-up and the corresponding negative control (lenti-Kv1.4-NC), containing enhanced green fluorescent protein (EGFP) were obtained from GeneChem (Shanghai). The viral titer was greater than 1 × 10^8^ TU.

### Isolation of TG neurons

Acute dissociation of TG neurons was performed as previously described [31–34]. Briefly, TGs were excised from ICR mice (male, 8 to 10 weeks old), cut into small pieces, and treated with 2.5 mg/ml collagenase D (Roche) for 35 min and 1.75 mg/ml trypsin for 20 min (Sigma). TG neurons were then mechanically dissociated by gentle trituration using narrow-bore fire-polished Pasteur pipettes. After trituration, the cells were centrifuged through a 15% bovine serum albumin gradient to remove cellular debris. After centrifugation, TG neurons were resuspended in B-27-supplemented Neurobasal-A medium (Thermo Fisher) and then seeded onto coverslips coated with Matrigel (2 mg/ml, BD Biosciences). Electrophysiological recordings were performed 3–7 h after plating.

### Electrophysiology

Patch clamp experiments were performed at room temperature using a standard whole-cell recording configuration as described previously [19, 35–37]. Briefly, whole-cell recordings were conducted using a MultiClamp 700B amplifier (Molecular Devices), and the output was digitized with a Digidata 1322 A converter (Molecular Devices). Data acquisition was achieved using pClamp 10.2, and patch clamp recording data were analyzed by Clampfit 10.2 software (Molecular Devices). Recording electrodes (resistance of 3 to 5 MΩ) were pulled from borosilicate glass microcapillary tubes (1.5-mm outer diameter, 0.86-mm inner diameter; World Precision Instruments) and filled with an internal solution containing (in mM) 140 KCl, 10 HEPES, 5 EGTA, 3 Mg-ATP, 1 MgCl_2_, 0.5 CaCl_2_, and 0.5 Na_2_GTP (pH = 7.4 and osmolality = 300 mosmol/kgH_2_O). The external solution for *I*_A_ recording contained (in mM) 150 choline-Cl, 10 HEPES, 10 glucose, 5 KCl, 1 MgCl_2_, and 0.03 CaCl_2_ (pH = 7.4 and osmolality = 305 mosmol/kgH_2_O). To separate the two kinetically different Kv currents in our whole-cell recordings, a large outward K^+^ current was evoked in small neurons by a command potential of + 40 mV from a holding potential of -80 mV. This typical current profile exhibited a rapid inactivation component and a subsequent sustained component. A short prepulse (150 ms) at -10 mV inactivated the *I*_A_, leaving only the *I*_DR_. Then, the subtraction of the *I*_DR_ from the total outward current yielded the *I*_A_. To obtain the current-voltage relationship of *I*_A_, neurons were held at -80 mV and stimulated with a series of depolarizing pulses ranging between − 70 and + 70 mV at 10-mV increments. To determine the voltage-dependent activation of *I*_A_, voltage steps of 400 ms were applied at 5 s intervals in + 10 mV increments from − 70 mV to + 70 mV. To determine the steady-state inactivation, conditioning prepulses ranging from − 120 mV to + 20 mV were applied at 5 s intervals in + 10 mV increments for 150 ms, followed by a 500 ms voltage step to + 40 mV. The pipette solution used for Ca^2+^ channel current recording contained (in mM) 110 CsCl, 25 HEPES, 10 EGTA, 4 Mg-ATP and 0.3 Na-GTP (pH = 7.4 and osmolality = 295 mosmol/kgH_2_O). The external solution used for Ca^2+^ channel current recording contained (in mM) 140 tetraethylammonium chloride, 10 HEPES, 5.5 glucose, 5 BaCl_2_, 5 CsCl and 0.5 MgCl_2_ (pH = 7.35 and osmolality = 300 mosmol/kgH_2_O). T-type channel currents were separated following the procedure described in our previous studies [30, 32]. The pipette solution used for Nav current recording and current clamp recording contained (in mM) 110 KCl, 25 HEPES, 10 NaCl, 4 Mg-ATP, 2 EGTA and 0.5 Na_2_GTP (pH = 7.3 and osmolality = 295 mosmol/kgH_2_O). The external solution contained (in mM) 128 NaCl, 30 glucose, 25 HEPES, 2 CaCl_2_, 2 MgCl_2_ and 2 KCl (pH = 7.4 and osmolality = 305 mosmol/kgH_2_O). To test neuronal excitability, TG neurons were held at *V*_rest_ and injected with 1-s depolarizing currents in 50-pA incremental steps until at least one action potential (AP) was elicited. We measured AP firing frequency (the number of spikes per second) in response to 150 pA depolarizing current injection in TG neurons before and after tyramine application. The first AP elicited using this paradigm was used to measure AP threshold, amplitude and first spike latency. In the present study, TG neurons were sorted into small (soma diameter < 25 μm) and medium-sized (soma diameter 25–35 μm) groups. We limited the whole-cell recording to the groups of small neurons, as they are primarily involved in the conduction and processing of nociception and pain [31, 37–40]. Compounds, including tyramine or vehicle, were puff-applied through a glass pipette using a pressure-pulsed microinjector (Picopump PV820). The tip was placed approximately 20 μm from the soma of TG neurons.

### Western blot analysis

Western blotting was performed as described previously [19, 26, 37, 41]. Briefly, TGs were homogenized in ice-cold RIPA lysis buffer (50 mM pH 7.4 Tris, 150 mM NaCl, 1 mM EDTA, 1% Triton X-100, 0.1% SDS and 1% sodium deoxycholate) supplemented with 1 mM phenylmethylsulphonyl fluoride, phosphatase inhibitors and protease inhibitor cocktail. Tissue lysates were prepared by sonication, incubated on a rocking platform, and then rotated at 4 °C before the supernatant was extracted. Equivalent amounts of extracted proteins (30 µg) were separated by 10% SDS–PAGE and electroblotted onto PVDF membranes (Merck Millipore). Membranes were blocked with 5% skim milk in TBST. Blotted proteins were probed with the following primary antibodies: anti-TAAR1 (rabbit, 1:1000, Thermo Fisher Scientific, catalog no. PA5-95704), anti-TAAR4 (rabbit, 1:1000, Novus Biologicals, catalog no. NBP3-10140), anti-Gαo (rabbit, 1:1000, Cell Signaling Technology, catalog no. #3975S), anti-Gαi (rabbit, 1:600, Cell Signaling Technology, catalog no. #5290S), anti-PKC_θ_ (rabbit, 1:500, Cell Signaling Technology, catalog no. #13,643), anti-Kv1.4 (rabbit, 1:800, Thermo Fisher Scientific, catalog no. PA5-85937), anti-Kv4.3 (rabbit, 1:600, Thermo Fisher Scientific, catalog no. PA5-95211) and anti-Kv3.4 (rabbit, 1:500, Thermo Fisher Scientific, catalog no. PA5-106236). An anti-β-actin antibody (rabbit, 1: 2000, Abcam, catalog no. ab8227) was used as the loading control. After washing three times with TBST, the membranes were incubated with a horseradish peroxidase-conjugated goat anti-rabbit (1:5000, Abcam, catalog no. ab6721) secondary antibody. Antibodies were validated by the manufacturers, and can be referred to datasheets of respective antibodies, which are listed in the supplementary materials (Table S1). Protein signals were visualized with an enhanced chemiluminescence (ECL) kit (Bio-Rad Laboratories). Images were acquired using a ChemiDoc XRS system and analyzed with Quantity One software (Bio-Rad Laboratories). Full-length blots are presented in the Supplementary Data.

### Subcellular fractionation

TG cells were treated with vehicle or 0.1 µM tyramine for 15 min. Cellular fractionation was conducted using a Subcellular Protein Fractionation Kit (Novus Biologicals) according to the manufacturer’s instructions. Protein concentrations were determined using a Bio-Rad Protein Assay. Fractionated proteins were analyzed by immunoblotting as described above.

### Reverse transcription-PCR (RT–PCR)

According to the standard procedure for TRIzol reagent (Invitrogen), total RNA was extracted from TG cells as described previously [42, 43] and was then quantified with a NanoDrop ND-1000 spectrophotometer (NanoDrop Technologies). After reverse transcription with a PrimeScript RT Reagent Kit (TaKaRa), a mixture of cDNA and specific primer pairs (RiboBio, Guangzhou, China) was subjected to PCR on an ABI VeritiPro PCR system. The sequences of the primers employed in this study are summarized in Table S2. All reactions were run in triplicate.

### Immunohistochemistry

Immuno**s**taining was performed as described previously [34, 37, 44]. In brief, tissue samples were sectioned (15 μm thickness) using a cryostat (CM1950, Leica Microsystems). Tissue sections were treated with 0.15% Triton X-100, blocked with 5% normal goat serum, and probed with primary antibodies against TAAR1 (rabbit, 1:300, Thermo Fisher Scientific, catalog no. PA5-115999), NeuN (mouse, 1:600, Cell Signaling Technology, catalog no. 94403s), GS (mouse, 1:1000, Abcam, catalog no. ab64613), CGRP (mouse, 1:500, Abcam, catalog no. ab81887) and NF200 (mouse, 1:500, Abcam, catalog no. ab215903). TG sections were subsequently visualized with Alexa Fluor 555-conjugated goat anti-rabbit IgG (1:300, Cell Signaling Technology, catalog no. 4413s), DyLight 488-conjugated goat anti-mouse IgG (1:300, Cell Signaling Technology, catalog no. 4408s), or IB4-fluorescein isothiocyanate (5 µg/ml; Sigma–Aldrich, catalog no. L2895). Antibodies are validated by the manufactures, and can be referred to datasheets of respective antibodies, which are listed in the Supplementary materials (Table S1). Images were acquired under an upright fluorescence microscope (Nikon 104 C) with a CoolSnap HQ2 CCD camera (Photometrics).

### Immunofluorescence analysis of translocation

Immunofluorescence analysis was performed as described previously [26]. Briefly, TG neurons were treated with tyramine for 15 min and then fixed with PFA (4%) in phosphate-buffered saline (PBS) for 20 min. The cells were sequentially permeabilized, blocked, and incubated with an anti-PKC_θ_ primary antibody (rabbit, 1:500, Cell Signaling Technology) for 16 h at 4 °C. After three washes in PBS, TG neurons were visualized with FITC-conjugated goat anti-rabbit IgG (Invitrogen, 1:600). Immunopositive signals were characterized using laser scanning confocal microscopy (Zeiss LSM 510, Germany) and analyzed with Image-Pro Plus analysis software (v.6.0; Media Cybernetics).

### Pharmacological agents

All chemicals were purchased from Sigma–Aldrich unless otherwise indicated. The QEHA (QEHAQEPERQYMHIGTMVEFAYALVGK) and SKEE peptides (SKEEKSDKERWQHLA DLADFALAMKDT) were synthesized by GenScript Corporation. Stock solutions of PTX, CTX, the epsilon-PKC specific inhibitor (εV1-2, Santa Cruz Biotechnology), the delta-PKC specific inhibitor (δV1-1, Santa Cruz Biotechnology), the PKC_η_ pseudosubstrate inhibitor (PKC_η_-PSI, Santa Cruz Biotechnology), the theta-PKC inhibitory peptide (PKC_θ_-IP, Santa Cruz Biotechnology), and AmmTx3 (Tocris Bioscience) were prepared with double deionized water (Milli-Q water systems, Merck). Stock solutions of tyramine, EPPTB, RO5263397, Gö6976 (Tocris Bioscience), KT-5720 (Calbiochem), chelerythrine chloride (Selleck), sotrastaurin (Selleck), CP339818 (Tocris Bioscience), and UK78282 were prepared in dimethyl sulfoxide (DMSO). The concentration of DMSO in each medium was less than 0.05% and had no significant effects on *I*_A_.

### Statistical analyses

Values are expressed as the means ± SEMs. No statistical method was used to predetermine sample sizes. Required experimental sample sizes were estimated based on previously established protocols in the field. The sample sizes were adequate as the differences between experimental groups were reproducible. All *n* values are clearly indicated within the figure legends. All statistical analyses were performed in GraphPad Prism 6.0 (Synergy Software) or SPSS 16.0 (SPSS) software. A paired *t* test was used to compare *I*_A_ from pre- and post- drug application, while an unpaired *t* test was used to compare two independent groups. Data were analyzed using one-way analysis of variance (ANOVA) followed by the Bonferroni *post hoc* test for multiple comparisons between groups. Two-way repeated-measures ANOVA with the Bonferroni *post hoc* test was used to analyze differences in behavioral test data. Differences with *p* < 0.05 were considered statistically significant.

## Results

### Tyramine selectively suppresses *I*_A_ in TG neurons

**Fig. 1:**
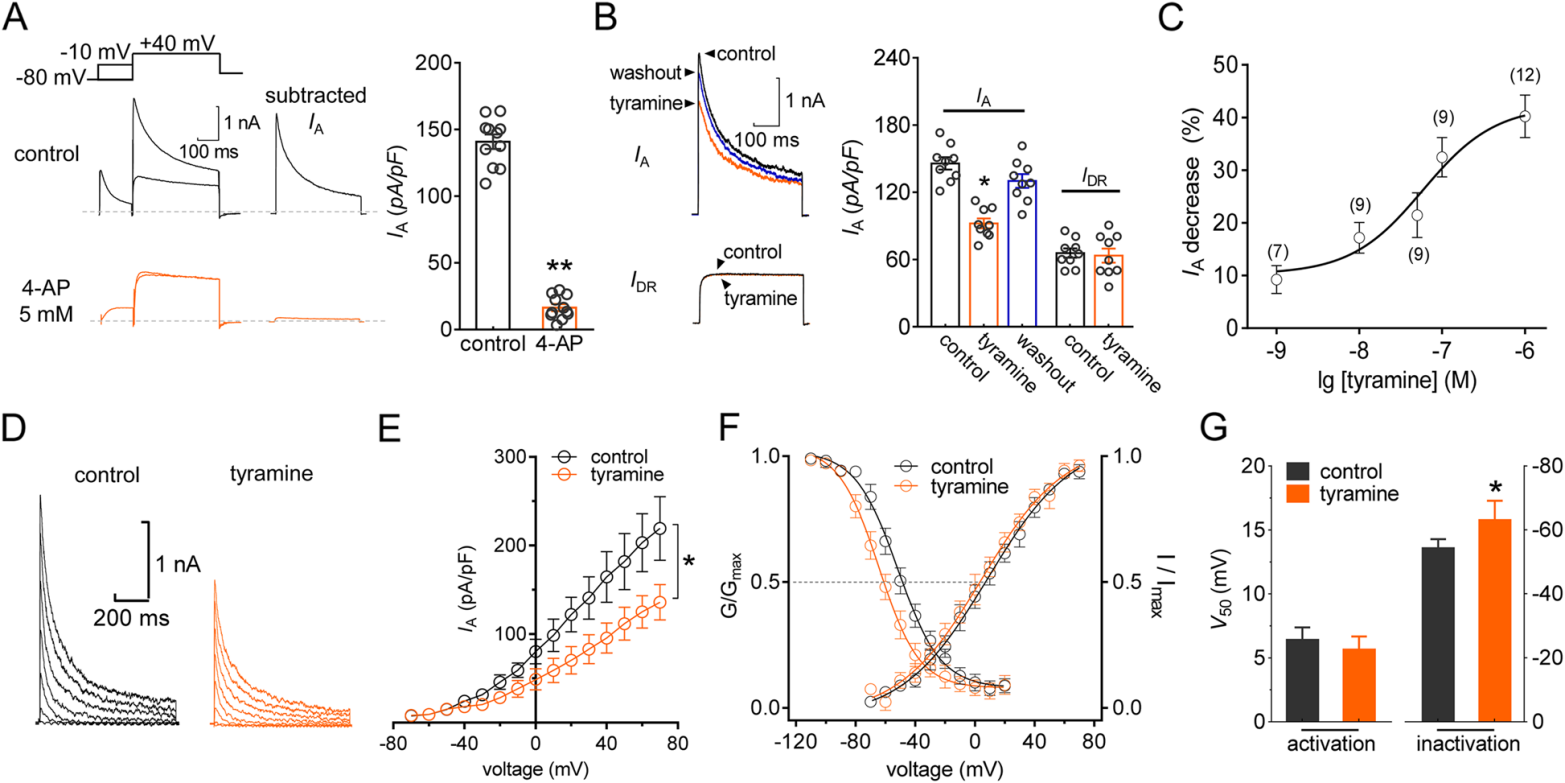
Tyramine suppresses *I*_A_ in TG neurons. **a** *I*_A_ isolation. *Left panel*, exemplary current traces before and after the application of 5 mM 4-aminopyridine (4-AP). *Insets*, remaining current after off-line subtraction of the noninactivating component of the current remaining after a brief prepulse to − 10 mV. *Right panel*, summary of results showing the effect of 5 mM 4-AP on *I*_A_ (*n* = 11 cells). **p < 0.01 vs. control, paired *t* test. **b** Exemplary current traces (*left panel*) and a bar chart (*right panel*) indicating that 0.1 µM tyramine selectively decreases *I*_A_ (*n* = 9 cells). *p < 0.05 vs. control, paired *t* test. **c**, The fitted dose–response curve showing tyramine-induced *I*_A_ inhibition. The IC_50_ was calculated from fits of dose–response data to the Hill equation. The numbers of TG neurons recorded at each tyramine concentration are indicated in round brackets. **d** and **e** Exemplary traces (*d*) and current-voltage (I-V) plots (*e*) of *I*_A_ in the absence and presence of 0.1 µM tyramine (*n* = 12 cells). *p < 0.05 vs. control, one-way ANOVA. **f** bath application of 0.1 µM tyramine did not affect the voltage-dependent activation curve (*n* = 11 cells) but shifted the steady-state inactivation curve leftward (*n* = 11 cells). Plots showing voltage-dependent activation and steady-state inactivation were fitted by the Boltzmann equation. **g** Bar chart summarizing the effect of tyramine on *V*_50_ in the activation or inactivation curve. *p < 0.05 vs. control, one-way ANOVA

Kv channels play crucial roles in regulating nociceptive responses by controlling neuronal excitability, and the related currents have been grouped into the transient outward *I*_A_ and the delayed rectifier *I*_DR_ in sensory neurons [14, 17, 45]. Therefore, we had to first separate the two kinetically distinct currents in small TG neurons (soma diameter < 25 μm), as they are the primary participants in peripheral nociceptive processing [30, 43, 46, 47]. Outward Kv currents were elicited from a holding potential of -80 mV to a test potential of + 40 mV. As demonstrated in Fig. 1A, typical currents exhibited a fast-inactivating transient component followed by slowly decaying and sustained components. A 150 msec prepulse to -10 mV was included to inactivate the transient channels, resulting in sustained *I*_DR_ isolation. Offline subtraction of *I*_DR_ from the total current yielded *I*_A_. This *I*_A_ was eliminated by bath application of 5 mM 4-AP (decrease of 89.3 ± 4.1%, *p* = 0.003, Fig. 1A), confirming effective *I*_A_ separation. Application of 0.1 µM tyramine to small TG neurons significantly reduced *I*_A_ by 32.6 ± 3.7% (*p* = 0.017), while *I*_DR_ remained unaffected (decrease of 2.6 ± 1.1%) (*p* = 0.822, Fig. 1B). The suppression of *I*_A_ was reversible after tyramine washout (Fig. 1B). This ability of tyramine to suppress *I*_A_ was further supported by the finding that tyramine decreased *I*_A_ in a dose-dependent manner (Fig. 1C). Confirmation by fitting to the Hill equation showed that the half-maximal inhibitory concentration (IC_50_) was 59.1 nM. We next characterized the biophysical mechanism of the *I*_A_ decrease induced by tyramine. The peak *I*_A_ amplitude was markedly reduced in response to 0.1 μm tyramine at all potentials above − 10 mV (*p* = 0.026, Fig. 1D and E). Moreover, tyramine had no significant effect on voltage-dependent activation properties (*V*_50_ from 6.5 ± 1.3 to 5.8 ± 1.1, *p* = 0.751, Fig. 1F and G) but shifted the half-maximal inactivation curve in a hyperpolarizing direction (*V*_50_ from − 53.9 ± 0.9 to 62.3 ± 1.8, *p* = 0.028, Fig. 1F and G).

### TAAR1 mediates the tyramine-induced *I*_A_ decrease

**Fig. 2:**
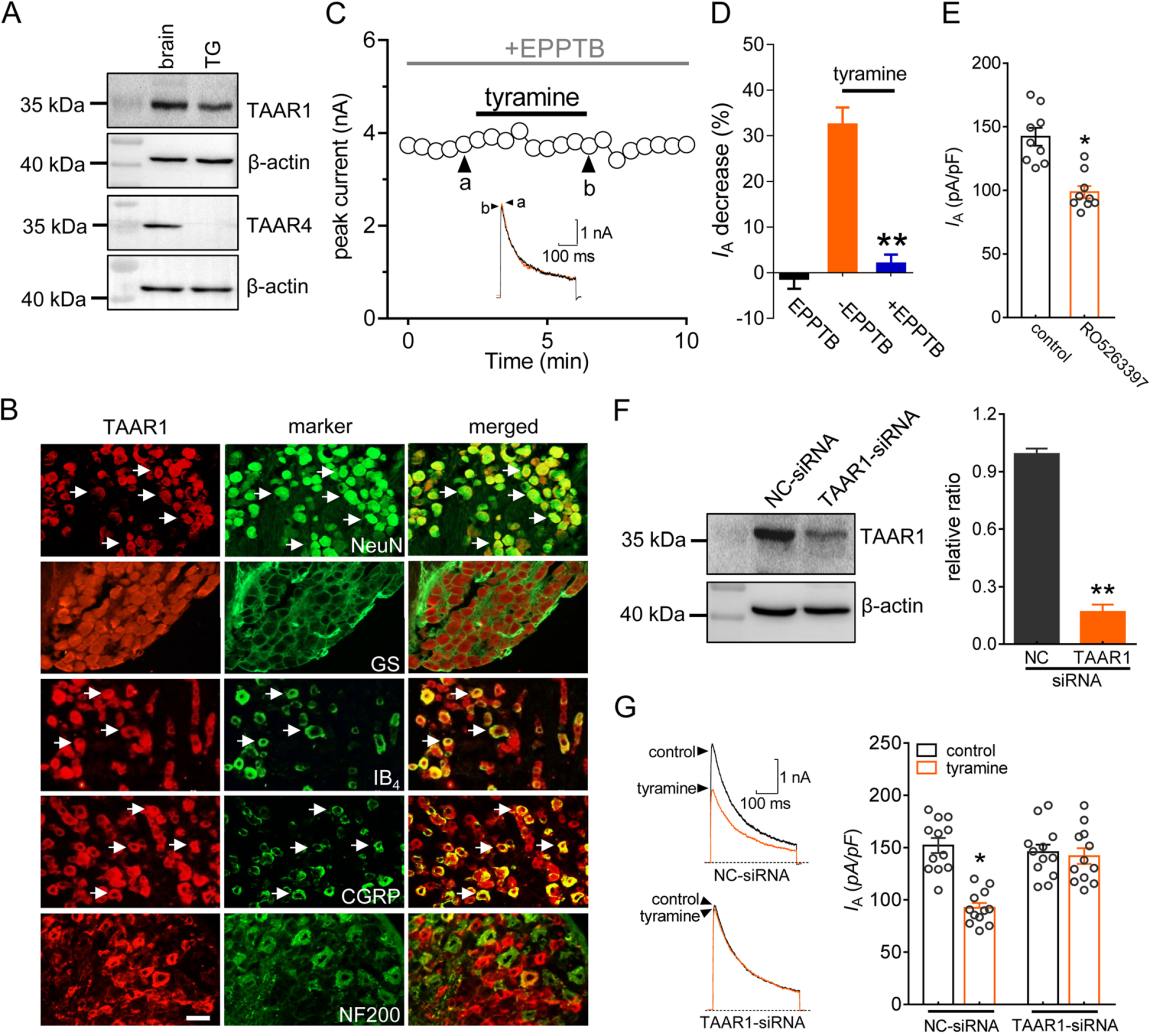
TAAR1 mediates the tyramine-induced reduction in *I*_A_. **a** Western blot analysis of TAAR1 and TAAR4 protein expression in mouse TGs. β-actin was used as an internal loading control. The immunoblots are representative of the results of at least three independent experiments. **b** Colocalization of TAAR1 (red) with NeuN, GS, IB_4_, CGRP or NF200 (green) in the TG (arrows). Scale bar = 50 μm. **c** and **d** The time course curve (*c*) and the bar chart (*d*) showing that pretreatment of TG neurons with EPPTB (3 µM) prevented the 0.1 µM tyramine-induced *I*_A_ decrease (*n* = 10 cells). EPPTB at 3 µM alone did not affect *I*_A_ (*n* = 7 cells). *Inset*s in panel *c* indicate the representative traces. The letters indicate the points used for sample traces. **p < 0.01 vs. tyramine, unpaired *t* test. **e** Summary of results indicating quantified *I*_A_ current density under control conditions, during exposure to RO5263397, and during washout (*n* = 9 cells). *p < 0.05 vs. control, paired *t* test. **f** Western blot analysis of TAAR1 protein expression in the NC-siRNA or TAAR1 siRNA-treated (TAAR1-siRNA) groups. The immunoblots are representative of the results of at least three independent experiments. **p < 0.01 vs. NC-siRNA, unpaired *t* test. **g** Representative traces (*left panel*) and bar chart (*right panel*) showing that treatment with TAAR1-siRNA abrogated the 0.1 µM tyramine-induced *I*_A_ response (*n* = 12 cells). *p < 0.05 vs. control + NC-siRNA, unpaired *t* test

TAARs belong to a class of G protein-coupled receptors that detect biogenic amines, and only TAAR1 and TAAR4 can be activated by trace amines [1, 2, 4, 48]. Thus, we examined whether these two receptors participate in the tyramine-induced *I*_A_ response. Western blot analysis of TG tissue lysates demonstrated that TAAR1 but not TAAR4 was endogenously expressed (Figs. 2A & S1). Immunostaining of intact TGs showed that TAAR1 was mostly colocalized with the neuronal marker NeuN but was also infrequently colocalized with glutamine synthetase (GS), a marker for satellite glial cells (Fig. 2B). Further double staining indicated that TAAR1 was expressed in a majority of peptidergic, calcitonin gene-related peptide (CGRP^+^)-containing neurons and a subset of nonpeptidergic, isolectin B4-binding (IB_4_^+^) nociceptive neurons but rarely in neurofilament 200 (NF200)-positive myelinated neurons (Fig. 2B). We next examined the participation of TAAR1 in the tyramine-mediated *I*_A_ decrease. When applied alone, EPPTB (3 µM), a selective antagonist of TAAR1, had no effect on *I*_A_ (decrease of -1.3 ± 2.2%), while pretreatment of TG neurons with EPPTB completely abolished the reduction in *I*_A_ induced by 0.1 µM tyramine (decrease of 2.1 ± 1.9%, *p* = 0.003, Fig. 2 C and D). Application of the TAAR1 agonist RO5263397 at 0.1 µM markedly reduced *I*_A_ in small TG neurons (decrease of 35.2 ± 3.7%, *p* = 0.02, Fig. 2E). To support this hypothesis, we further examined the effect of tyramine on *I*_A_ in TAAR1-silenced TG neurons. Intra-TG injection of a chemically modified siRNA led to highly efficient uptake in mouse TG cells (Fig. S2). Western blot analysis demonstrated that intra-TG administration of TAAR1-siRNA markedly decreased the protein abundance of TAAR1 in TG cells (*p* = 0.006, Figs. 2F & S3). Knockdown of TAAR1 abrogated 0.1 µM tyramine-mediated *I*_A_ suppression in small TG neurons (decrease of 29.9 ± 2.1% in NC-siRNA, *p* = 0.026; decrease of 2.3 ± 0.9% in TAAR1-siRNA, *p* = 0.391) (Fig. 2G).

### The TAAR1 response requires the G_βγ_-dependent novel PKC isoform

**Fig. 3:**
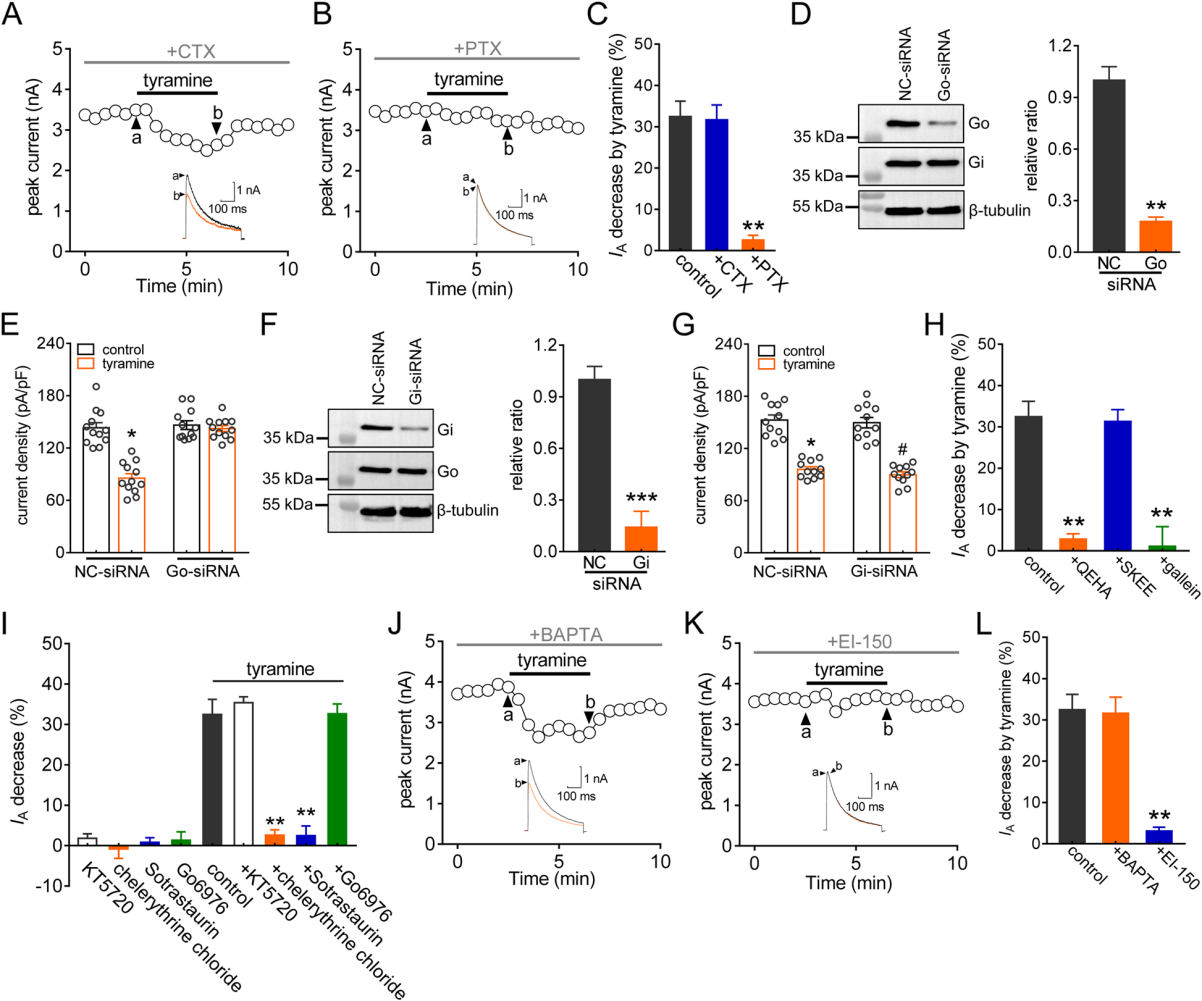
The TAAR1-mediated *I*_A_ suppression requires novel PKC isozymes. **a** and **b** The time course curve of *I*_A_ changes induced by tyramine (0.1 µM) in TG neurons pretreated with cholera toxin (CTX, 0.5 µg/mL for 16 h, *n* = 9 cells, *a*) or pertussis toxin (PTX, 0.2 µg/ml for 16 h, *n* = 10 cells, *b*). *Inset*s indicate the representative traces. The letters indicate the points used for sample traces. **c** Summary data showing the effect of tyramine on *I*_A_ in TG neurons pre-incubated with CTX or PTX. **p < 0.01 vs. control, paired *t* test. **d** Protein expression of G_o_ or G_i_ in TG cells treated with NC-siRNA or G_o_ siRNA (G_o_-siRNA). The immunoblots are representative of the results of at least three independent experiments. **p < 0.01 vs. control, unpaired *t* test. **e** Summary data demonstrating that G_o_-siRNA treatment abrogated the 0.1 µM tyramine-induced *I*_A_ response (*n* = 12 cells). *p < 0.05 vs. control + NC-siRNA, unpaired *t* test. **f** Protein expression of G_i_ or G_o_ in TG cells treated with NC-siRNA or G_i_-siRNA. The immunoblots are representative of the results of at least three independent experiments. ***p < 0.001 vs. NC-siRNA, unpaired *t* test. **g** Summary of results demonstrating that G_i_-siRNA treatment did not affect the ability of tyramine (0.1 µM) to decrease *I*_A_ (*n* = 11 cells). *p < 0.05 vs. control + NC-siRNA, ^#^*p* < 0.05 vs. control + G_i_-siRNA, unpaired *t* test. **h** Summary data demonstrating the effect of 0.1 µM tyramine on *I*_A_ in cells dialyzed with 10 µM QEHA (*n* = 8 cells), 10 µM SKEE (*n* = 11 cells) or 10 µM gallein (*n* = 9 cells). **i** Summary data demonstrating the effect of 0.1 µM tyramine on *I*_A_ in TG neurons pre-incubated with KT5720 (1 µM, *n* = 10 cells), chelerythrine chloride (1 µM, *n* = 11 cells), sotrastaurin (100 nM, *n* = 10 cells) or Go6976 (200 nM, *n* = 11 cells). Application of 1 µM KT5720 (*n* = 6 cells), 1 µM chelerythrine chloride (*n* = 6 cells), 100 nM sotrastaurin (*n* = 5 cells), or 200 nM Go6976 (*n* = 7 cells) alone did not affect *I*_A_. **p < 0.01 vs. control, paired *t* test. **j** and **k** The time course curve of *I*_A_ changes induced by tyramine (0.1 µM) in small TG neurons dialyzed with BAPTA (20 µM, *n* = 11 cells, *j*) or EI-150 (100 µM, *n* = 10 cells, *k*). *Inset*s indicate the representative traces. The letters indicate the points used for sample traces. **l** Summary data demonstrating the effect of 0.1 µM tyramine on *I*_A_ indicated in panels *j* and *k*. **p < 0.01 vs. control, paired *t* test

TAAR1 activation triggers the accumulation of intracellular cAMP and then modulates PKA signaling via G protein signaling or interferes with β-arrestin2 signaling via G protein-independent mechanisms [48–50]. Thus, we examined whether—and if so, which—G proteins participate in the TAAR1-mediated *I*_A_ decrease. Pretreatment with cholera toxin (500 ng/mL), which inactivates Gs by ADP ribosylation, did not affect the ability of tyramine to reduce *I*_A_ (decrease of 32.7 ± 3.6%, *p* = 0.833, Fig. 3A and C). Conversely, inhibition of heterotrimeric G_i/o_ proteins by pretreatment of cells with 200 ng/ml pertussis toxin (PTX) completely prevented the tyramine-mediated *I*_A_ response (decrease of 2.6 ± 1.2%, *p* = 0.008, Fig. 3B and C), indicating that G_i/o_ but not G_s_ is involved in the tyramine-induced reduction in *I*_A_. To determine which G_i_ and G_o_ might be involved, we individually knocked down G_o_ or G_i_ in TG cells by a chemically modified siRNA. Compared to that in control siRNA-treated cells, the protein abundance of G_o_ was markedly decreased in TG cells transduced with G_o_-siRNA, whereas the G_i_ protein expression level remained unchanged (*p* = 0.006, Figs. 3D & S4). G_o_-siRNA transduction prevented the 0.1 µM tyramine-induced *I*_A_ response (decrease of 31.1 ± 2.3% in NC-siRNA, *p* = 0.016; decrease of 1.9 ± 0.5% in G_o_-siRNA, *p* = 0.915) (Fig. 3E). In contrast, treatment of TG cells with G_i_-siRNA decreased the protein abundance of G_i_ (*p* < 0.001, Figs. 3F & S5), while the tyramine-induced decrease in *I*_A_ was not affected in cells transduced with G_i_-siRNA (decrease of 34.9 ± 3.9% in NC-siRNA, *p* = 0.03; decrease of 36.1 ± 4.2% in G_i_-siRNA, *p* = 0.022; Fig. 3G). We further determined the implication of the βγ subunit (G_βγ_) of G_o_ in the TAAR1-induced reduction in *I*_A_. Intracellular infusion of the G_βγ_ inhibitory peptide QEHA (10 µM) prevented the tyramine-induced decrease in *I*_A_ (decrease of 2.8 ± 1.2%, *p* = 0.003, Fig. 3H), while the scrambled peptide SKEE (10 µM) elicited no such effect (decrease of 31.3 ± 2.8%, *p* = 0.575, Fig. 3H). Similar findings were observed with another G_βγ_ inhibitor, gallein. Pretreatment of TG neurons with 10 µM gallein abrogated the tyramine-induced *I*_A_ response (decrease of 1.3 ± 1.6%, *p* = 0.008, Fig. 3H). Previous studies have suggested the participation of protein kinase A (PKA) in *I*_A_ regulation [47]. However, preincubation of TG neurons with the PKA inhibitor KT-5720 (1 µM) did not alter the ability of tyramine to decrease *I*_A_ (decrease of 35.3 ± 1.5%, *p* = 0.785, Fig. 3I). PKC has also been shown to modulate *I*_A_ [46], and it can be a downstream effector of G_βγ_ activation [51]. Pretreatment of TG neurons with the inhibitor of both conventional and novel PKCs chelerythrine chloride (1 µM) abolished the *I*_A_ reduction induced by 0.1 µM tyramine (decrease of 2.9 ± 1.1%, *p* = 0.002, Fig. 3I). Similar results were observed with sotrastaurin (100 nM), another potent inhibitor of novel and conventional PKCs (decrease of 2.3 ± 1.7%, *p* = 0.003, Fig. 3I). In contrast, pretreatment of TG neurons with Gö6976 (200 nM), an inhibitor specific for conventional PKCs (decrease of 32.6 ± 2.5%, *p* = 0.619, Fig. 3I), elicited no such effect. In contrast to conventional PKC isoforms, the novel isoforms (δ, ϵ, η, θ) of PKC bind to DAG but are independent of Ca^2+^. Intracellular infusion of the fast Ca^2+^ chelator BAPTA (20 µM) via the recording pipette solution did not affect the tyramine-mediated *I*_A_ decrease (decrease of 31.6 ± 3.9%, *p* = 0.813, Fig. 3J and L), while pretreatment of cells with EI-150 (100 µM), a DAG antagonist, abrogated the tyramine-induced *I*_A_ response (decrease of 3.2 ± 0.7%, *p* = 0.005, Fig. 3K and L), further supporting the involvement of novel PKC isoforms.

### PKC _θ_ is involved in the TAAR1-mediated I_A_ response

**Fig. 4:**
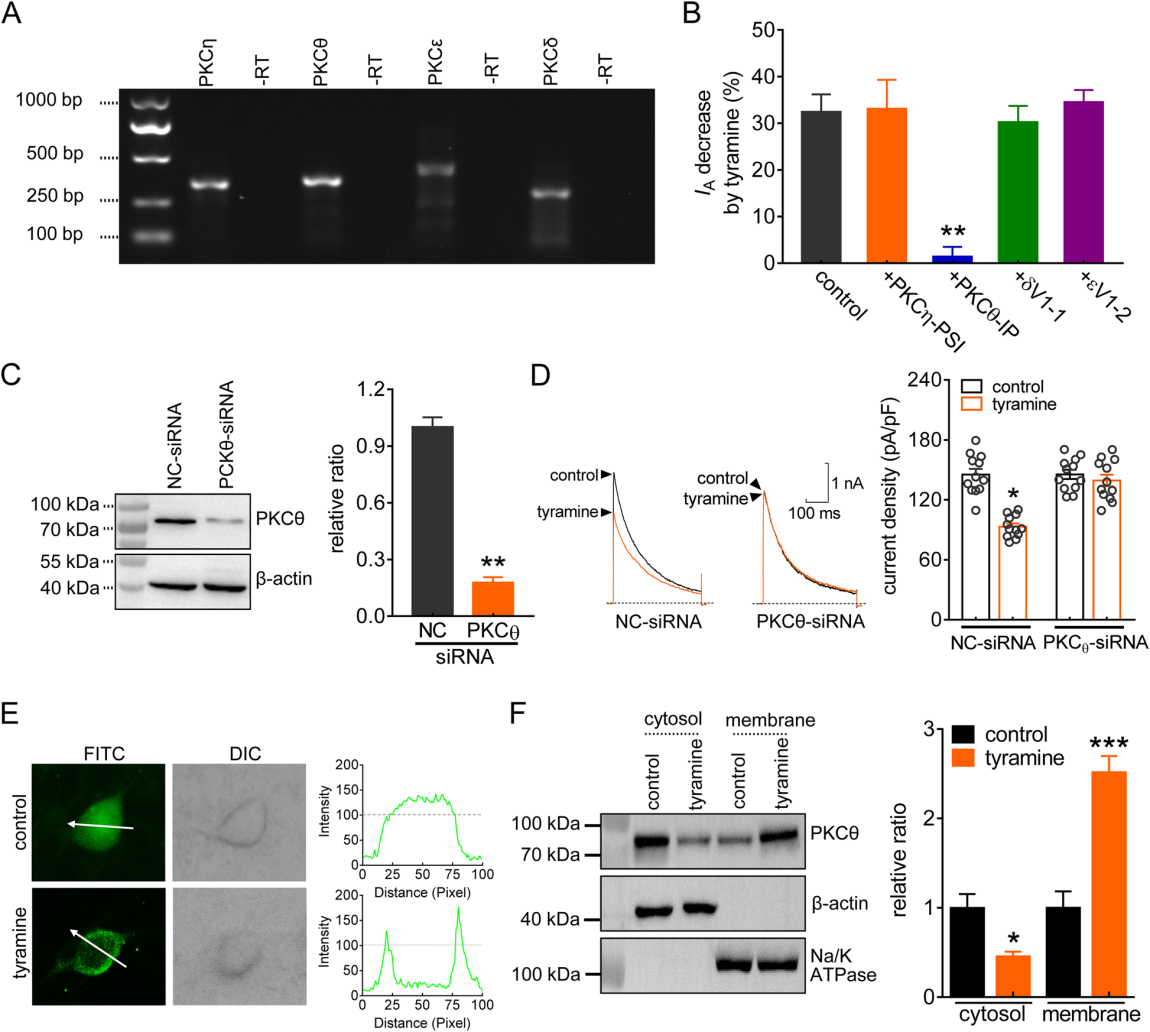
PKC_θ_ is involved in the TAAR1-mediated *I*_A_ response. **a** The expression of transcripts of novel PKC isozymes (θ, ε, δ and η) in mouse TGs. Samples without reverse transcriptase (-RT) were used as negative controls. **b** Summary data demonstrating the effect of tyramine (0.1 µM) on *I*_A_ in small TG neurons dialyzed with PKC_η_-PSI (10 µM, *n* = 9 cells), PKC_θ_-IP (10 µM, *n* = 9 cells), δV1-1 (1 µM, *n* = 10 cells) or εV1-2 (2 µM, *n* = 11 cells). **p < 0.01 vs. control, paired *t* test. **c** Western blot analysis of PKC_θ_ protein abundance in TG cells treated with NC-siRNA or PKC_θ_-siRNA. The immunoblots are representative of the results of at least three independent experiments. **p < 0.05 vs. NC-siRNA, unpaired *t* test. **d** Representative traces (*left panel*) and summary data (*right panel*) showing that PKC_θ_-siRNA treatment abrogated the 0.1 µM tyramine-induced *I*_A_ response (*n* = 12 cells). Tyramine at 0.1 µM significantly decreased *I*_A_ in NC-siRNA-treated TG neurons (*n* = 12 cells). *p < 0.05 vs. control + NC-siRNA, unpaired *t* test. **e** Immunofluorescence analysis of PKC_θ_ translocation mediated by 0.1 µM tyramine. The white arrows indicate the line-scanned area. Data are representative of three separate experiments. **f** Western blot analysis of PKC_θ_ expression in cytoplasmic and membrane fractions isolated from TG cells treated with 0.1 µM tyramine. α-Na^+^/K^+^ ATPase served as an indicator for membrane contamination of cytosolic extracts. β-actin was used as a control for protein loading. The immunoblots are representative of the results of at least three independent experiments. **p < 0.01 vs. control, unpaired *t* test

We next identified the exact novel PKC isoforms involved in the response to tyramine. RT–PCR analysis revealed that transcripts of all four novel PKC isoforms—i.e., PKC_η_, PKC_θ_, PKC_ε_, and PKC_δ_—were present in mouse TGs (Figs. 4A & S6). Dialysis of neurons with the PKC_θ_ inhibitory peptide (PKC_θ_-IP, 10 µM) completely abolished the TAAR1-mediated *I*_A_ response (decrease of 1.5 ± 2.1%, *p* = 0.006, Fig. 4B), while no such effects were elicited by dialysis with PKC_η_-PSI (10 µM), a PKC_η_ pseudosubstrate inhibitor; δV1-1 (1 µM), a peptide inhibitor of the delta isoform; or εV1-2 (2 µM), a specific inhibitor of the epsilon isoform (decreases of 33.1 ± 6.2% for PKC_η_-PSI, *p* = 0.515; 30.3 ± 3.4% for δV1-1, *p* = 0.398; 34.6 ± 2.5% for εV1-2, *p* = 0.661; Fig. 4B). To further confirm that the signaling was PKC_θ_-dependent, we knocked down PKCθ expression in TG cells. Compared to that in TG cells treated with control siRNA, the protein abundance of PKC_θ_ was markedly decreased in PKC_θ_-siRNA-treated cells (*p* = 0.002, Figs. 4C & S7). PKC_θ_ knockdown eliminated the tyramine-mediated *I*_A_ response (decrease of 31.3 ± 2.7% in NC-siRNA, *p* = 0.027; decrease of 3.3 ± 2.7% in PKC_θ_-siRNA, *p* = 0.325) (Fig. 4D). As activation of PKC_θ_ leads to its translocation from the cytosol to the plasma membrane, we evaluated the translocation of PKC_θ_ in small TG neurons treated with tyramine. Immunofluorescence labeling demonstrated that 0.1 µM tyramine clearly induced PKC_θ_ recruitment to the membrane of the soma (*p* = 0.039, Fig. 4E). Consistent with this finding, 0.1 µM tyramine did not affect total PKC_θ_ protein expression in TG cells (Fig. S8) but induced a significant increase in membrane-bound PKC_θ_ and a decrease in the cytosolic fraction (*p* < 0.001, Figs. 4F & S9).

### Activation of TAAR1 decreases *I*_A_ by targeting Kv1.4 channels

**Fig. 5:**
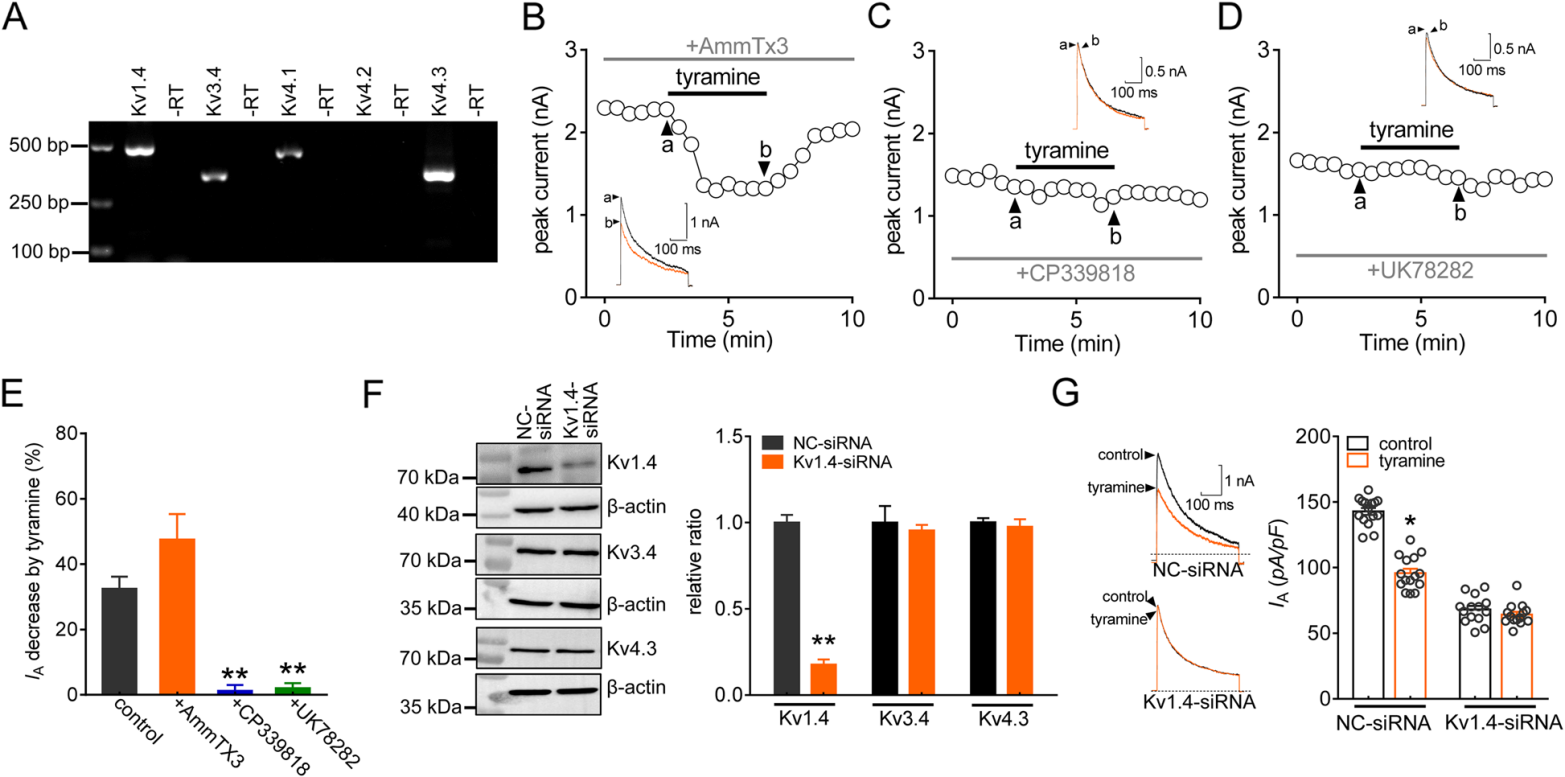
Activation of TAAR1 decreases *I*_A_ by targeting Kv1.4 channels. **a** The expression of Kv1.4, Kv3.4, Kv4.1, Kv4.2 and Kv4.3 transcripts in mouse TGs. Samples without reverse transcriptase (-RT) were used as negative controls. **b** through **d** The time course curves of *I*_A_ changes induced by 0.1 µM tyramine in the presence of AmmTX3 (2 µM, *n* = 11 cells, *b*), CP339818 (1 µM, *n* = 9 cells, *c*) or UK78282 (1 µM, *n* = 10 cells, *d*). *Inset*s in each panel indicate the representative traces. The letters indicate the points used for sample traces. **e** Summary data indicating the effect of tyramine on *I*_A_ in the presence of AmmTX3, CP339818 or UK78282. **p < 0.01 vs. control, paired *t* test. **f** Western blot analysis demonstrating the protein abundance of Kv1.4, Kv3.4 or Kv4.3 in the control siRNA (NC-siRNA) and Kv1.4-siRNA-treated (Kv1.4-siRNA) groups. β-actin served as a loading control. The immunoblots are representative of the results of at least three independent experiments. **p < 0.01 vs. NC-siRNA, unpaired *t* test. **g** Representative traces (*left panel*) and bar graph (*right panel*) demonstrating that Kv1.4-siRNA treatment abrogated the tyramine-mediated *I*_A_ reduction (*n* = 14 cells). Tyramine at 0.1 µM significantly decreased *I*_A_ in cells transduced with NC-siRNA (*n* = 15 cells). *p < 0.05 vs. control + NC-siRNA, unpaired *t* test

It has been established that the Kv1.4, Kv3.4, Kv4.1, Kv4.2, and Kv4.3 subunits may contribute to *I*_A_ in sensory neurons [15, 21, 52, 53]. We therefore determined whether—and if so, which—subunits of these Kv channels mediate PKC_θ_-dependent *I*_A_ regulation. The expression of these channel subunits in mouse TGs was first examined. RT–PCR analysis demonstrated that both Kv1.4 and Kv4.3 were expressed abundantly in mouse TGs, while the mRNA levels of Kv4.1 and Kv3.4 were relatively low. No Kv4.2 mRNA signal was detected (Figs. 5A & S10). We further identified the Kv subunits participating in the tyramine-mediated *I*_A_ response. Tyramine (0.1 µM) still robustly decreased *I*_A_ (by 47.5 ± 7.8%, *p* = 0.861, Fig. 5B and E) in cells pretreated with AmmTX3 (2 µM), a selective blocker of Kv4 channels. Interestingly, the application of tyramine to small TG neurons failed to affect *I*_A_ (decrease of 1.2 ± 1.8%, *p* = 0.003, Fig. 5 C and E) when the cells were preincubated with CP339818 (1 µM), a potent Kv1.3 and Kv1.4 channel blocker. Similar results were obtained with another Kv1.4 channel blocker, UK78282 (1 µM; decrease of 2.1 ± 1.5%, *p* = 0.008, Fig. 5D and E), demonstrating that tyramine might decrease *I*_A_ by targeting Kv1.4 channels. As complementary support for this hypothesis, we employed a siRNA knockdown approach to reduce the expression of Kv1.4. Intra-TG injection of chemically modified Kv1.4-siRNA decreased the protein expression level of Kv1.4 in TG cells (*p* = 0.006, Figs. 5F & S11), whereas the levels of Kv3.4 (*p* = 0.572) and Kv4.3 remained unchanged (*p* = 0.739, Figs. 5F & S11). Treatment with Kv1.4-siRNA completely prevented the tyramine-induced *I*_A_ decrease (decrease of 32.5 ± 3.6% in NC-siRNA, *p* = 0.027; decrease of 3.7 ± 1.9% in Kv1.4-siRNA, *p* = 0.883; Fig. 5G).

### TAAR1 enhances TG neuronal excitability

**Fig. 6:**
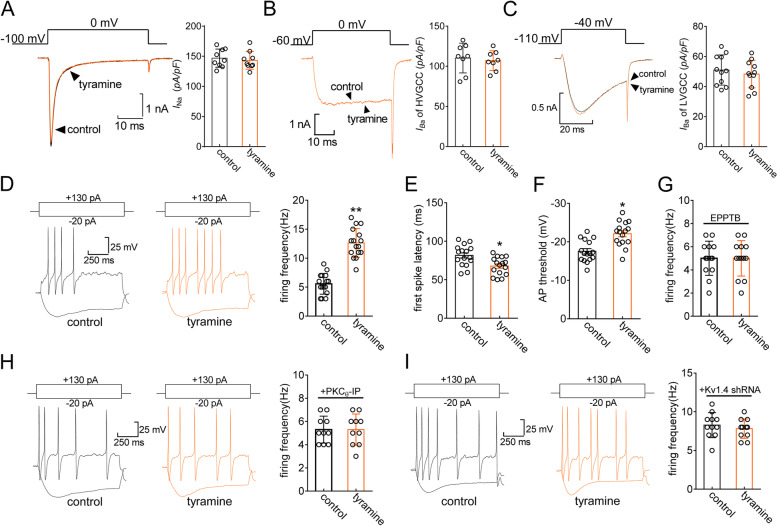
TAAR1 increases membrane excitability in TG neurons. **a** through **c** Representative traces (*right panel*) and summary data (*right panel*) demonstrating that 0.1 µM tyramine had no effects on Na^+^ channel currents (*n* = 9 cells, *a*), high-voltage-activated Ca^2+^ channel currents after 2 µM ω-conotoxin-GVIA treatment (*n* = 8 cells, *b*), or low-voltage-activated T-type Ca^2+^ channel currents (*n* = 11 cells, *c*) in small TG neurons. **d** Representative traces (*left panel*) and summary data (*right panel*) indicating that 0.1 µM tyramine decreased the action potential (AP) firing rate (*n* = 16 cells). **p < 0.01 vs. control, paired *t* test. **e** and **f** Tyramine at 0.1 µM shortened the first-spike latency (*E, n* = 16 cells) and lowered the AP threshold (*F*, *n* = 16 cells). *p < 0.05 vs. control, paired *t* test. **g** Summary data demonstrating that EPPTB (3 µM) treatment prevented the tyramine-induced increase in the AP firing rate (*n* = 13 cells). **h** and **i** Representative traces (*left panel*) and summary data (*right panel*) demonstrating that treatment with either PKC_θ_-siRNA (*n* = 10 cells, *h*) or Kv1.4-siRNA (*n* = 11 cells, *i*) abolished the tyramine-induced increase in the AP firing rate

We further examined whether tyramine affects TG neuronal excitability, in which *I*_A_ plays critical roles [16, 54]. Tyramine (0.1 µM) did not affect Nav currents (decrease of 2.5 ± 0.8%, *p* = 0.912, Fig. 6A) in small TG neurons. It has been demonstrated that tyramine might inhibit presynaptic N-type Ca^2+^ channels in substantia gelatinosa neurons [55]. After adding 2 µM ω-conotoxin-GVIA to the external solution to block N-type channels, we found that tyramine had no further effect on the remaining high-voltage-activated Ca^2+^ channel currents (decrease of 3.1 ± 1.1%, *p* = 0.726, Fig. 6B). Furthermore, tyramine (0.1 µM) did not affect low-voltage-activated (T-type) Ca^2+^ channel currents (decrease of 3.5 ± 1.9%, *p* = 0.577, Fig. 6C). Therefore, using a bath solution containing 2 µM ω-conotoxin-GVIA, we found that tyramine (0.1 µM) markedly increased the action potential firing rate (122.8 ± 10.6%, *p* = 0.007, Fig. 6D). Moreover, the application of tyramine decreased the first-spike latency (*p* = 0.033, Fig. 6E) and lowered the threshold (*p* = 0.019, Fig. 6F). The tyramine-induced increase in the potential firing rate was abrogated by pretreatment of TG neurons with 3 µM EPPTB (*p* = 0.615, Fig. 6G) and by dialysis of neurons with PKC_θ_-IP (10 µM) (*p* = 0.852, Fig. 6H). In addition, Kv1.4-siRNA treatment abrogated the tyramine-induced increase in the action potential firing rate (*p* = 0.773, Fig. 6I).

### TAAR1 mediates nociceptive behaviors

**Fig. 7:**
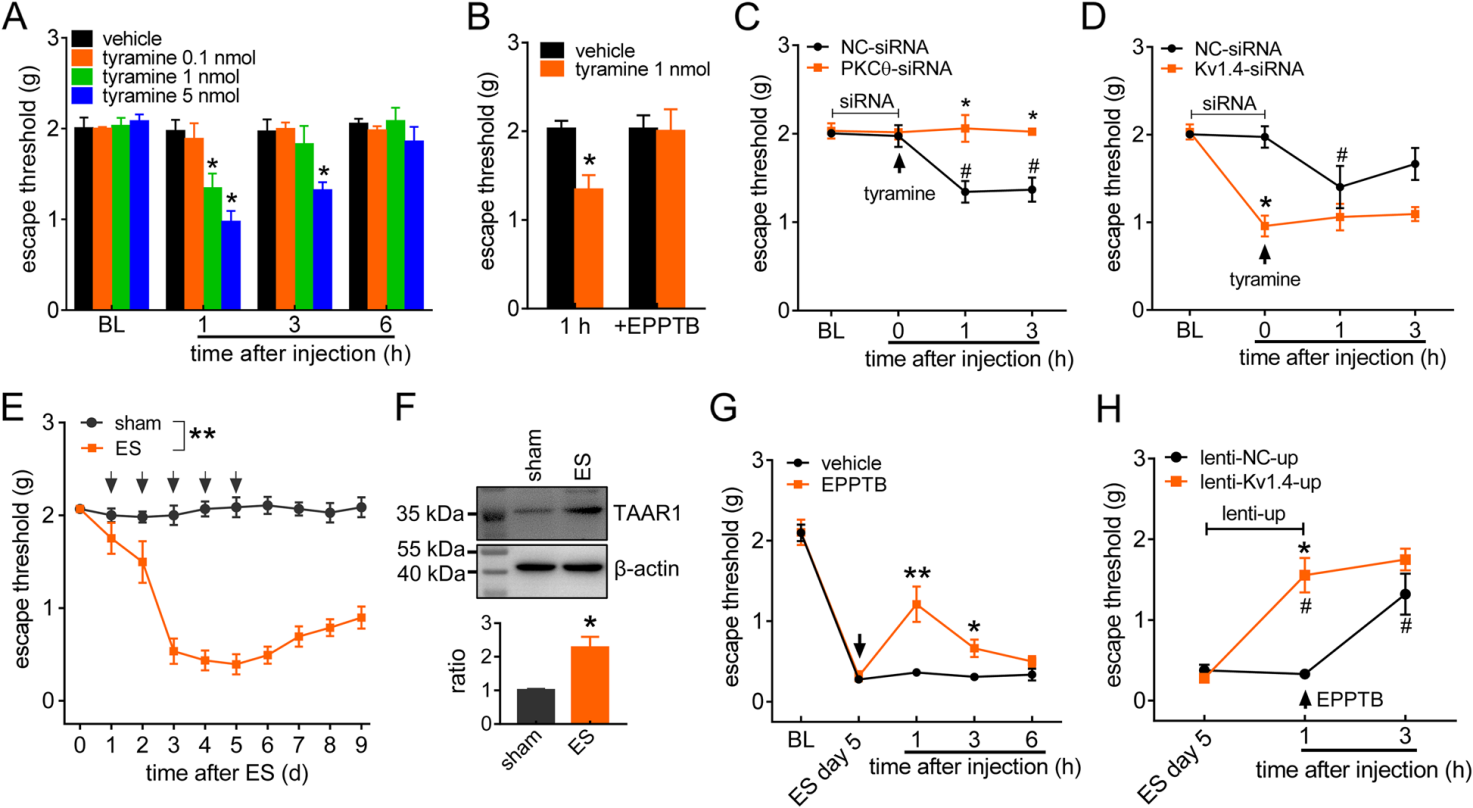
Peripheral TAAR1 contributes to mechanical pain hypersensitivity in mice. **a** Escape threshold after intra-TG injection of vehicle or tyramine at 0.1 nmol, 1 nmol or 5 nmol. *p < 0.05 vs. vehicle at the corresponding time point, two-way ANOVA. BL, baseline. **b** Intra-TG pre-injection of EPPTB (3 nmol) prevented 1 nmol tyramine-induced mechanical hypersensitivity. *p < 0.05 vs. vehicle at 1 h, one-way ANOVA. **c**, Effects of PKC_θ_-siRNA or the control siRNA (NC-siRNA) on tyramine (1 nmol, intra-TG injection; arrow)-induced mechanical hypersensitivity of acute pain. *p < 0.05 vs. NC-siRNA at the corresponding time point, ^#^*p* < 0.05 vs. NC-siRNA at 0 h, two-way ANOVA. **d** Intra-TG pre-injection of Kv1.4-siRNA occluded 1 nmol tyramine (arrow)-induced mechanical hypersensitivity. *p < 0.05 vs. NC-siRNA at 0 h, ^#^*p* < 0.05 vs. NC-siRNA at 0 h, two-way ANOVA. **e** Escape threshold to mechanical stimuli in the sham- and ES-operated groups. **p < 0.01 vs. sham, two-way ANOVA. **f** Western blot analysis of TAAR1 protein abundance in the sham- and ES (day 5)-operated groups. *p < 0.05 vs. sham, unpaired *t* test. The immunoblots are representative of the results of at least three independent experiments. **g** Intra-TG injection of EPPTB (3 nmol, arrow) alleviated mechanical allodynia on day 5 post-ES. *p < 0.05, **p < 0.01 vs. vehicle at the corresponding time point, two-way ANOVA. *H*, Intra-TG injection of lenti-Kv1.4-up occluded the EPPTB (3 nmol, arrow)-induced alleviation of mechanical allodynia in ES 5 d mice. *p < 0.05 vs. ES 5 d, ^#^*p* < 0.05 vs. lenti-NC-up at 1 h, two-way ANOVA. At least 8 mice were used per experimental group in all behavior experiments

We further determined the contribution of TAAR1 signaling to the nociceptive response at the whole-animal level. Compared with the corresponding vehicle group, intra-TG injection of 1 nmol (*p* = 0.039) or 5 nmol tyramine at 1 h (*p* = 0.017) markedly enhanced acute pain sensitivity to mechanical stimuli, while 0.1 nmol tyramine did not elicit such effects (*p* = 0.433). The effect of tyramine at 5 nmol was maintained at 3 h (*p* = 0.02) and recovered at 6 h (Fig. 7A). The tyramine-mediated mechanical pain hypersensitivity was abrogated by prior intra-TG application of 5 nmol EPPTB (*p* = 0.823, Fig. 7B). To further validate PKC_θ_ and Kv1.4 as important molecular targets for pain hypersensitivity via tyramine/TAAR1 signaling, we individually applied chemically modified siRNAs specific for PKC_θ_ or Kv1.4. As indicated in Fig. 7C, compared to the control siRNA (NC-siRNA), intra-TG administration of PKC_θ_-siRNA reversed 1 nmol tyramine-induced mechanical pain hypersensitivity (*p* = 0.013 at 1 h, *p* = 0.015 at 3 h; Fig. 7C). Administration of tyramine at 1 nmol still increased mechanical pain sensitivity in the NC-siRNA-transduced groups (*p* = 0.027 at 1 h, *p* = 0.02 at 3 h; Fig. 7C). The participation of Kv1.4 channels in the tyramine-mediated nociceptive response was also examined. Compared to NC-siRNA, Kv1.4-siRNA exhibited a significant increase in mechanical sensitivity after intra-TG administration (*p* = 0.03 at 0 h; Fig. 7D). Further administration of 1 nmol tyramine significantly increased mechanical pain sensitivity in the NC-siRNA group (*p* = 0.031, Fig. 7D), while injection of tyramine had no additive effect with Kv1.4-siRNA on mechanical pain sensitivity (Fig. 7D). These results indicated that Kv1.4 is involved in the response to tyramine in vivo.

Next, we examined the possible role of TAAR1 signaling in a mouse model of migraine. Compared to sham-operated groups, mice subjected to electrical stimulation (ES) of the dura mater surrounding the superior sagittal sinus exhibited significant mechanical allodynia (*p* = 0.008, Fig. 7E). Moreover, immunoblot analysis of TG protein lysates at Day 5 post-ES, the time point of peak mechanical allodynia, revealed a significant increase in protein expression of TAAR1 compared to that after sham surgery (*p* = 0.03, Figs. 7F & S12). Other time-points on Day 1 through Day 5 were also tested. The protein expression of TAAR1 in ES-treated mice was significantly increased on Day 3 and was maintained on Day 5 (Fig. S13), while there were no significant differences in mice following sham surgery (Fig. S13).

Thus, we measured the effects of TAAR1 blockade on ES-induced mechanical allodynia and found that intra-TG application of EPPTB (3 nmol) attenuated mechanical allodynia on Day 5 post-ES (*p* = 0.008 at 1 h and *p* = 0.03 at 3 h) (Fig. 7G). This effect lasted for at least 3 h. Furthermore, to validate the Kv1.4 channel as a pivotal target for the migraine pain-relieving response of TAAR1 signaling, we induced Kv1.4 expression specifically in TG neurons using a neuron promoter (human synapsin 1 gene promoter)-specific combinatorial lentiviral vector lenti-hSyn-Kv1.4-up (lenti-Kv1.4-up) containing enhanced green fluorescent protein (EGFP) as an expression marker. Local overexpression of Kv1.4 in TG neurons 5 days after ES significantly alleviated mechanical allodynia compared with the control (lenti-NC-up) (*p* = 0.02, Fig. 7H). Intra-TG administration of EPPTB (3 nmol) in lenti-Kv1.4-up-treated mice had no further effect on mechanical sensitivity, as evidenced by the stability of the escape threshold throughout the 3-h period after EPPTB application (*p* = 0.522, Fig. 7H). In contrast, intra-TG injection of EPPTB induced a significant increase in the escape threshold in mice treated with lenti-Kv1.4-NC (*p* = 0.03, Fig. 7H). Collectively, these findings suggested that Kv1.4 participated in TAAR1-mediated pain sensitivity in an ES model of migraine.

## Discussion

**Fig. 8:**
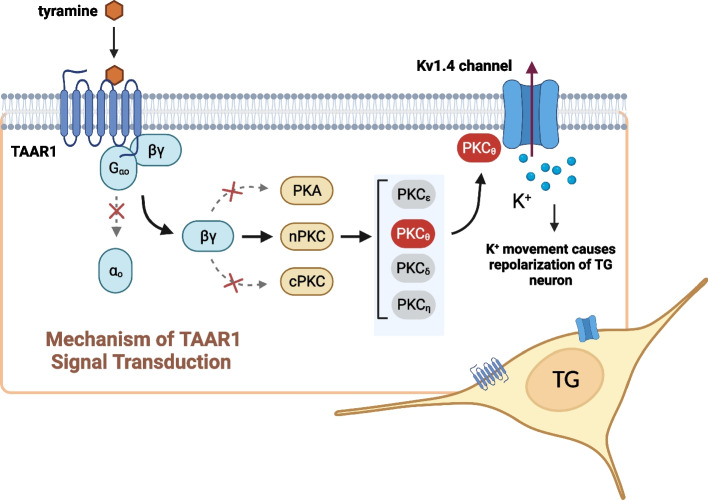
The proposed mechanisms by which TAAR1 regulates Kv1.4 channels in TG neurons. Tyramine acts through Go protein-coupled TAAR1, leading to the release of G_βγ_ subunits. The G_βγ_ dimer stimulates the downstream nPKC_θ_, which selectively modulates Kv1.4 channel activity, resulting in *I*_A_ suppression. The signaling cascade mediated by TAAR1 contributes to TG neuronal hyperexcitability and the nociceptive behaviors of tyramine. Neither PKA nor conventional PKC isoforms are involved in the tyramine-mediated *I*_A_ response. Nevertheless, whether nPKC_θ_ phosphorylates Kv1.4 channels directly or acts via some intermediate signaling molecules needs to be further explored

In this study, we provided mechanistic insight into a critical functional role of tyramine in selectively inhibiting Kv1.4-mediated *I*_A_ in trigeminal ganglion neurons, with *I*_DR_ remaining unaffected. The hyperpolarizing shift of *V*_50,inact_ suggested that the increased proportion of A-type channels in the steady-state inactivation might be one of the factors responsible for the tyramine-induced decrease in *I*_A_. Our studies showed that this effect is mediated by TAAR1 coupling to the G_o_ protein, leading to the release of G_βγ_ and triggering the activation of downstream PKC_θ_ signaling (see Fig. 8 for a schematic diagram illustrating the proposed mechanism). Physiologically, this TAAR1-mediated signaling contributes to sensory neuronal hyperexcitability and the nociceptive response to tyramine in mice.

TAAR1 can couple to the G_s_ protein, resulting in the stimulation of adenylate cyclase and PKA [50]; couple to the promiscuous G_q/13_ protein, resulting in mobilization of intracellular Ca^2+^ [56]; or interfere with the β-arrestin2-mediated pathway via G protein-independent mechanisms [48]. Surprisingly, such mechanisms are unlikely to be involved in TG neurons, as indicated in the present study. We provided evidence of G_i/o_ participation in the TAAR1-mediated *I*_A_ response in mouse TG neurons, since pretreatment with PTX but not CTX abolished the response to tyramine. Distinct from the G_i_ protein, which regulates adenylyl cyclase, the G_o_ protein mediates its action through G_βγ_ dimers [35, 57, 58]. In TG neurons, we identified that the G_βγ_ subunit of the PTX-sensitive G_o_ protein participated in the TAAR1-mediated decrease in *I*_A_ because (1) siRNA-mediated knockdown of G_o_ completely abolished the tyramine-induced *I*_A_ response and (2) intracellular application of G_βγ_ inhibitors abrogated the response to tyramine. However, it is still unclear how G_βγ_ regulates Kv1.4 channel activity. G_βγ_ has been suggested to interact directly with G protein-gated K^+^ channels [59]. However, this mechanism does not seem to be reproduced in Kv1.4 channels in TG neurons, since the TAAR1-mediated *I*_A_ response was further blocked by inhibition of downstream protein kinases. G_βγ_ might stimulate PKA to regulate various molecular targets, including potassium channels [35, 60]. In superficial dorsal horn neurons, stimulation of PKA was found to significantly reduce *I*_A_ [47]. Similarly, activation of PKA by forskolin reduced the peak amplitude of *I*_A_ in rat hippocampal CA1 neurons [46] as well as in zebrafish white skeletal muscle fibers [61]. In contrast, a PKA inducer mimicking the effect of neuromedin U receptors, which enhanced *I*_A_ in dorsal root ganglion neurons, has been reported [35]. However, in this study, we found that the tyramine-induced *I*_A_ response was not affected by PKA regulation, indicating that mechanisms other than PKA signaling are involved. We propose that the tyramine/TAAR1 interaction decreases *I*_A_ in small TG neurons via PKC_θ_-dependent signaling. Activation of PKC is associated with its intracellular translocation from the cytoplasm to the plasma membrane. This can occur rapidly at room temperature. For instance, C-type natriuretic peptide can induce PKC translocation to the plasma membrane [62]. Additionally, in dorsal root ganglion neurons, treatment with insulin-like growth factor causes the translocation of PKCα from the cytosol to the membrane [37]. These findings support the potential involvement of PKC_θ_ in the TAAR1–mediated *I*_A_ suppression observed in TG neurons. Consistent with the present study, another study showed that activation of metabotropic glutamate receptors results in *I*_A_ inhibition in striatal cholinergic interneurons through PKC signaling [63]. Similar results have been reported in superficial dorsal horn neurons [47] and in CA1 pyramidal neurons [64]. However, studies on the role of PKC signaling in regulating *I*_A_ channels have produced conflicting results [46, 65]. For instance, in large aspiny neurons in the striatum, activation of PKC_α_ upregulated *I*_A_ after ischemia [65], while interestingly, inhibition of *I*_A_ by urotensin-II receptor through PKC_α_ in sensory neurons has also been reported [54]. Although the discrepancies need further investigation, the differential regulatory effects of PKC on *I*_A_ may vary in tissues/cell types expressing distinct PKC isozymes [66, 67]. Second, PKC-interacting proteins, conferring specificity on individual PKC isoforms, endow different isoforms with the ability to perform specific cellular functions [68]. Third, distinct splice variants of the accessory β subunits of Kv1.4 channels may produce different modulatory effects on *I*_A_ [69, 70]. Parenthetically, we cannot rule out the possibility that some intermediate proteins recruited by distinct PKC isoforms can participate in the tyramine-mediated *I*_A_ response.

Alterations in the membrane excitability of peripheral sensory neurons can directly affect nociceptive behaviors [11, 12]. *I*_A_ is a key component that regulates neuronal excitability and has been implicated in the control of both spike frequency and first-spike latency [16, 71], which are the two important determinants of temporal neurotransmitter release [72]. Both pharmacological and genetic studies have established that peripheral *I*_A_ modulation affects nociceptive somatic inputs and mediates neuropathy in various neuropathic pain models [17]. Consistent with the tyramine-induced *I*_A_ decrease, stimulation of TAAR1 in TG neurons markedly increased the firing frequency along with the reduction in first-spike latency. In addition, the nociceptive effects of TAAR1 are mediated partially, if not completely, through its inhibitory effects on Kv1.4 channels in TG neurons. Indeed, it has been demonstrated that Kv1.4-encoded *I*_A_ channels in mature cortical pyramidal neurons contribute to the repetitive firing rate [73], although recordings from cortical pyramidal neurons lacking both Kv4.2 and Kv4.3 revealed that Kv1.4 encodes a minor component of *I*_A_ [74]. Moreover, blocking Kv1.4 channels could substantially prolong axonal action potentials via a reduction in their repolarization slope in midbrain dopamine neurons [75]. In line with our present study, previous in vivo studies have demonstrated that the application of TAAR1 agonists induced pronociceptive effects [7]. Ingestion of foods containing high levels of tyrosine by patients taking monoamine oxidase inhibitors produces headaches and chest pain [5]. Importantly, clinical evidence supports the hypothesis that headache patients had higher blood levels of tyramine [8]. However, in some double-blinded human studies, tyramine was not found to induce migraine attacks in patients with migraine or headache in healthy controls, compared to placebo [76, 77]. Interestingly, in an open-label study indoramin reportedly “blocked” tyramine induced migraine attacks [78]. Worth to note, in this paper, these attacks developed very late (> 24 h) after intravenous administration of tyramine and much later than usual triggered migraine attacks (< 12 h), raising the possibility that these migraine attacks were spontaneous. These discrepancies may be attributed to the fact that these studies are from before the development of the International Classification Headache Disorders. In addition, potential channel targets other than A-type channels could be involved in TAAR1 signaling, a possibility that needs further investigation. For instance, it has been suggested that tyramine acts presynaptically to decrease the expression of N-type Ca^2+^ channels [55]. Moreover, tyramine produced voltage-independent potentiation of recombinant ASIC1a in transiently transfected CHO cells but not of native ASICs in hippocampal interneurons [79]. In addition, although we introduced a strict control group (vehicle or sham surgery) to demonstrate the contribution of tyramine-mediated signaling to mechanical pain hypersensitivity, there is a limitation in this study due to the lack of an extracephalic region used as a control.

## Conclusion

Collectively, this study presents new insights into the effect of tyramine on Kv1.4 channels in nociceptive sensory neurons. Our results suggest that tyramine reduces Kv1.4-mediated *I*_A_ by stimulating G_o_ protein-coupled TAAR1 and downstream G_βγ_-dependent PKC_θ_ signaling. The identified signaling initiated by tyramine mediates TG neuronal hyperexcitability and mechanical pain hypersensitivity in mice. Considering the above mentioned findings in humans, it would be interesting to tease out the differences in PKC_θ_ and Kv1.4 signaling between model animals and humans, and examine whether headache disorders such as migraine can be further divided into subgroups based on distinct signature signaling pathways. Such investigations would provide opportunities for novel therapeutic strategies for the treatment of headache disorders.

## Supplementary Information

**Additional file 1.**

## Data Availability

All data and materials generated in this study are available upon request.

## Abbreviations

TAAR1: trace amine-associated receptor 1
TG: trigeminal ganglion
Kv: voltage-gated K^+^ channels
*I*_A_: transient outward K^+^ channel currents
*I*_DR_: sustained delayed-rectifier K^+^ channel currents
4-AP: 4-aminopyridine
CGRP: calcitonin gene related peptide
PKA: protein kinase A
PKC: protein kinase C
