# Supplementary material for: Trace amine-associated receptor 1 regulation of Kv1.4 channels in trigeminal ganglion neurons contributes to nociceptive behaviors
Source: J Headache Pain. 2023 May 8;24(1):49. doi: 10.1186/s10194-023-01582-5 (PMC10165857; doi:10.1186/s10194-023-01582-5)
Supplement: Supplementary file 1 — Additional file 1. [file 10194_2023_1582_MOESM1_ESM.pdf]

Supplementary data

Trace amine-associated receptor 1 regulation of Kv1.4 channels in trigeminal ganglion neurons contributes to nociceptive behaviors

Yuan Zhang [1, 3, 4, #, *], Hua Wang [2, #], Yufang Sun [3, #], Zitong Huang [3], Yu Tao [3], Yiru Wang [1], Xinghong Jiang [3], Jin Tao [3, 4, *]

[1] Department of Geriatrics & Clinical Research Center of Neurological Disease, The Second Affiliated Hospital of Soochow University, Suzhou 215004, P.R. China;

[2] Department of Endocrinology, Shanghai East Hospital, Tongji University School of Medicine, Shanghai 200120, P.R. China;

[3] Department of Physiology and Neurobiology & Centre for Ion Channelopathy, Medical College of Soochow University, Suzhou 215123, P.R. China;

[4] Jiangsu Key Laboratory of Neuropsychiatric Diseases, Soochow University, Suzhou 215123, P.R. China;

[#] These authors contribute equally to this work.

*To whom correspondence should be addressed:

Dr. Yuan Zhang, Department of Geriatrics & Clinical Research Center of Neurological Disease, The Second Affiliated Hospital of Soochow University, 1055 San-Xiang Road, Suzhou 215004, P.R. China. E-mail: yuanzhang@suda.edu.cn

Dr. Jin Tao, Department of Physiology and Neurobiology & Centre for Ion Channelopathy, Medical College of Soochow University, 199 Ren-Ai Road, Suzhou 215123, P.R. China. E-mail: taoj@suda.edu.cn

This PDF file includes: Figures S1 to S13 and Tables S1 to S2.

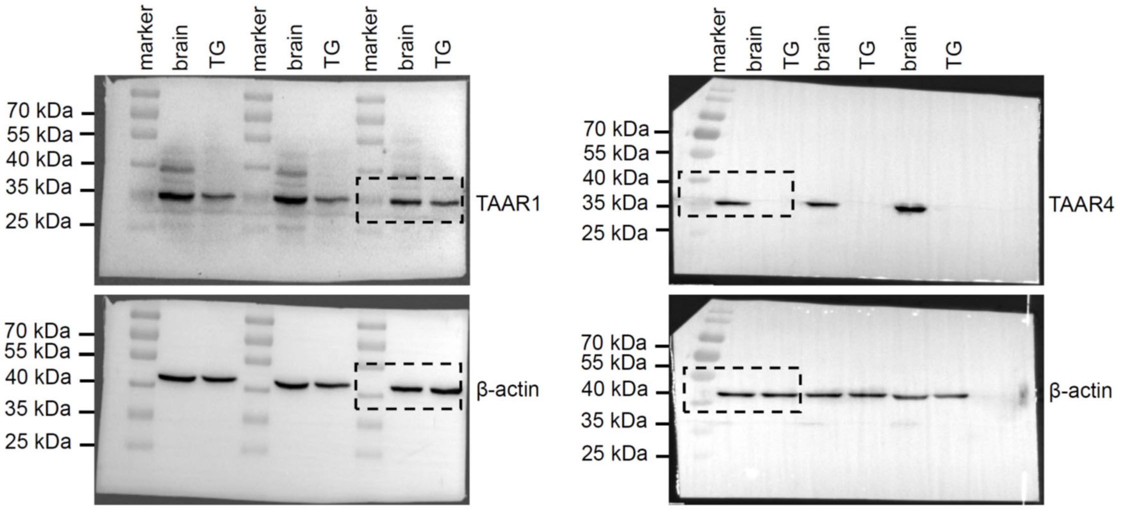

Figure S1: Immunoblots showing the protein expression of TAAR1 and TAAR4 in mouse TGs. Shown are the expanded images of Western blots presented in Fig. 2A. Blots are representative of three experiments.

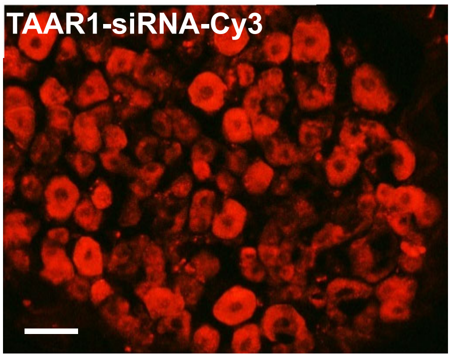

Figure S2: Representative image of Cy3 expression with red fluorescence in an intact TG 3 days after intra-TG injection of a 5'-cholesteryl-modified and 2'-O-methyl-modified TAAR1-siRNA labeled with Cy3. Scale bar, 50 µm.

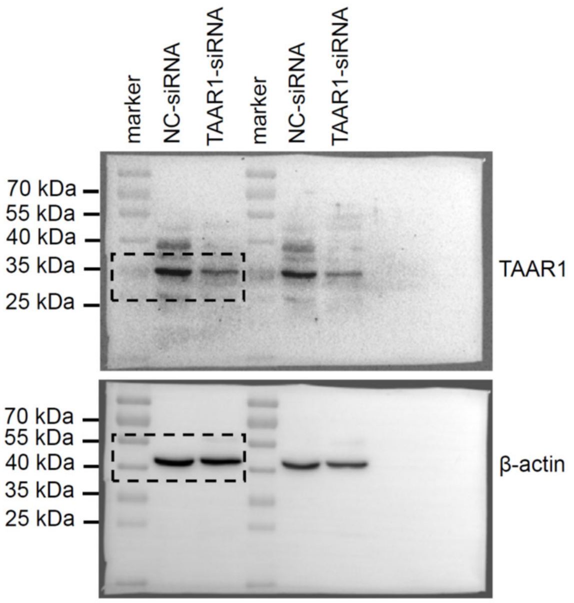

Figure S3: The protein expression of TAAR1 was attenuated by intra-TG injection of TAAR1-siRNA. Shown is the expanded image of Western blot for TAAR1 against a loading control, β-actin, presented in Fig. 2F. Blots are representative of three experiments.

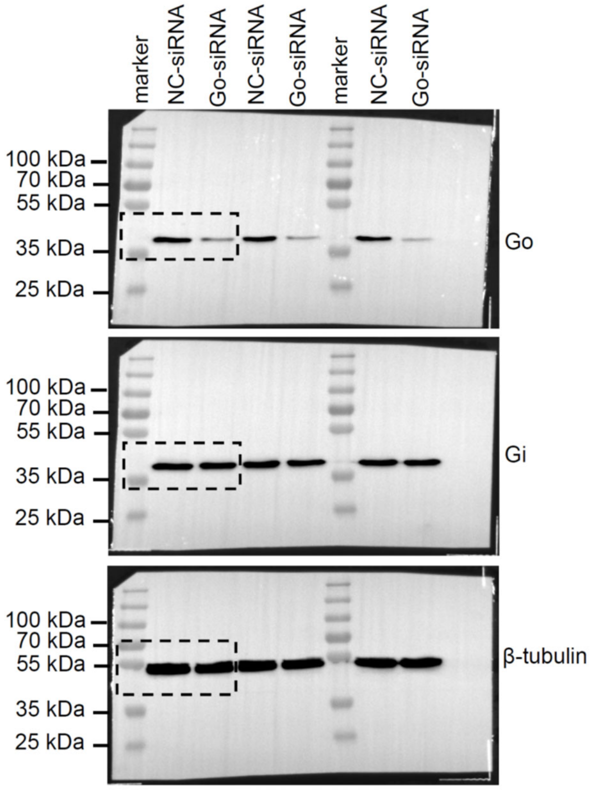

Figure S4: Intra-TG injection of Go-siRNA decreased the protein expression of Gαo, but not Gαi. Shown is the expanded image of Western blot for Go and Gi against a loading control, β-tubulin, presented in Fig. 3D. Blots are representative of three experiments.

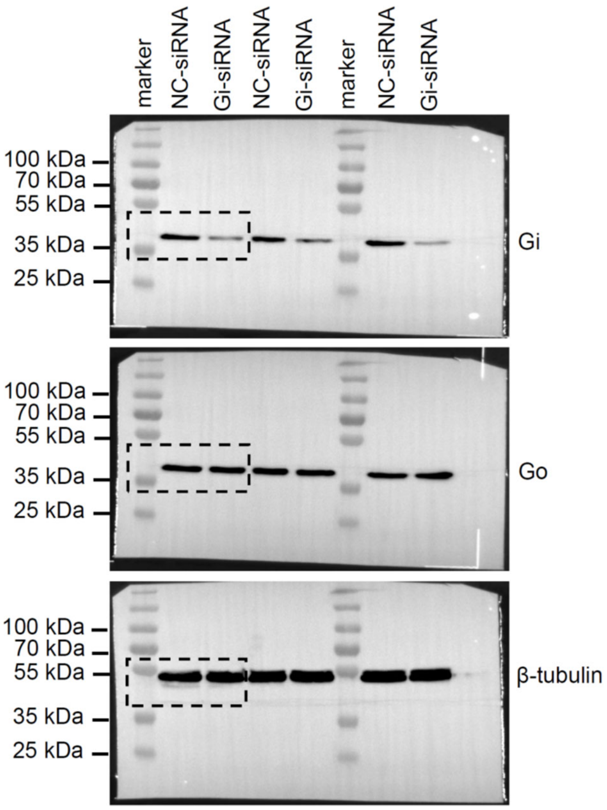

Figure S5: Intra-TG injection of Gi-siRNA decreased the protein expression of Gαi, but not Gαo. Shown is the expanded image of Western blot for Gi and Go against a loading control, β-tubulin, presented in Fig. 3F. Blots are representative of three experiments.

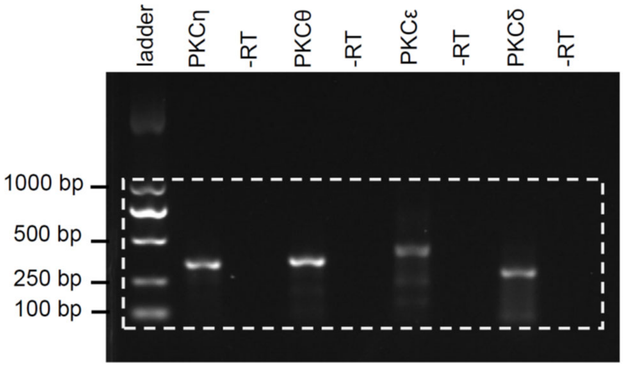

Figure S6: RT-PCR analysis of PKC isoforms in the TGs of naïve mice. Shown is the expanded image of RT-PCR for four PKC isoforms (η, θ, ε, and δ) presented in Fig. 4A.

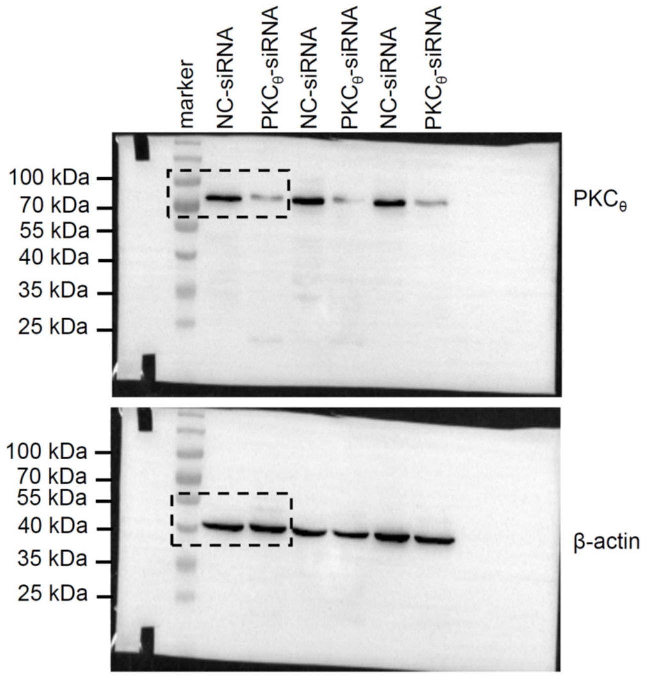

Figure S7: The protein expression of PKC$_θ$ was attenuated by intra-TG injection of PKC$_θ$-siRNA. Shown is the expanded image of Western blot for PKC$_θ$ against a loading control, β-actin, presented in Fig. 4C. Blots are representative of three experiments.

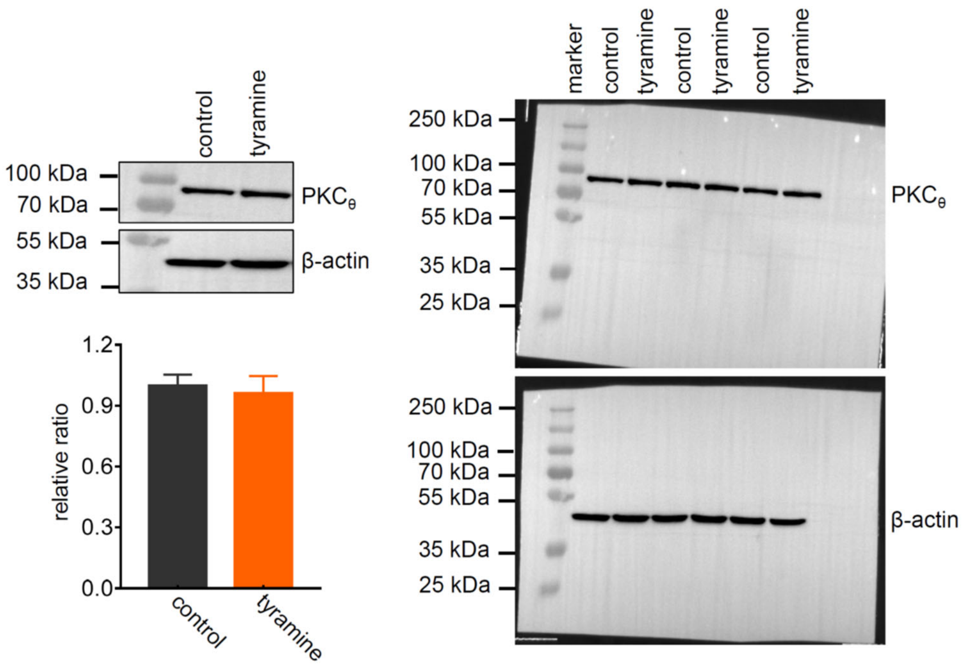

Figure S8: Tyramine at 0.1 μM did not affect the protein abundance of PKC$_θ$ in TG cells. Shown are the full-length blots for PKC$_θ$ against a loading control, β-actin. Blots are representative of three experiments.

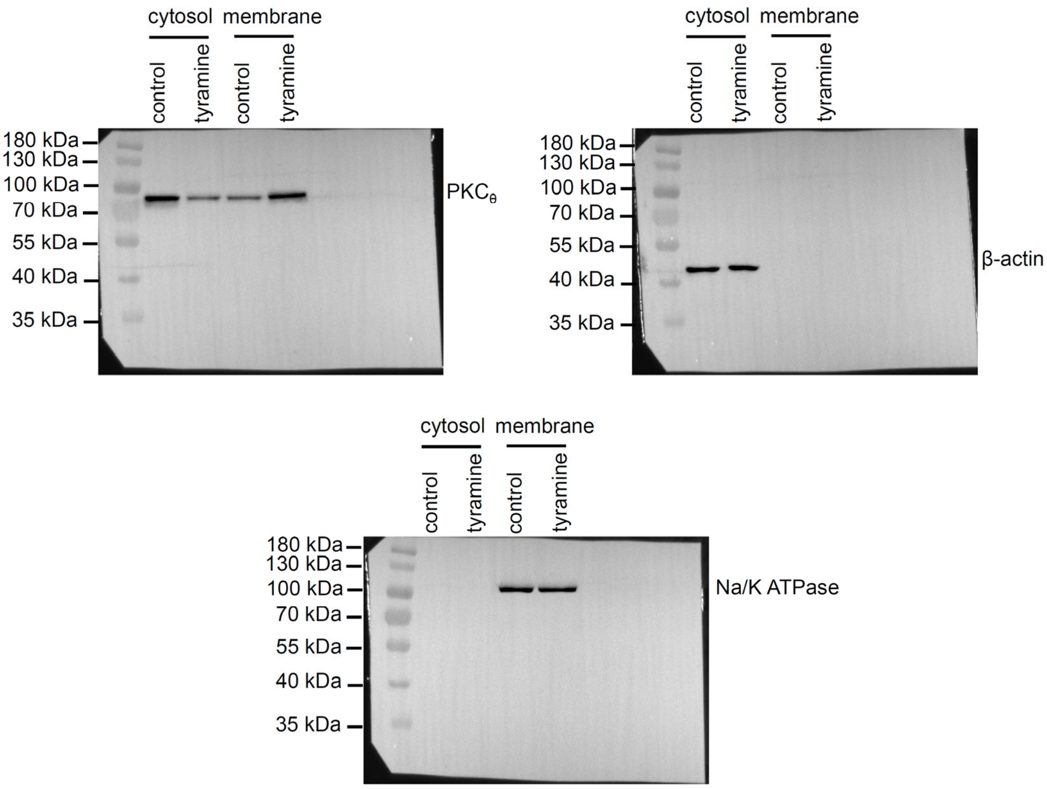

Figure S9: Tyramine at 0.1 μM drives PKC_θ **cytoplasm-to-membrane translocation.** TG cells were untreated or treated with tyramine for 15 min before being processed for the cellular compartment fractionation assay. α-Na^+/K^+ ATPase served as an indicator for membrane contamination of cytosolic extracts. β-actin was used as a control for protein loading. Blots are representative of three experiments.

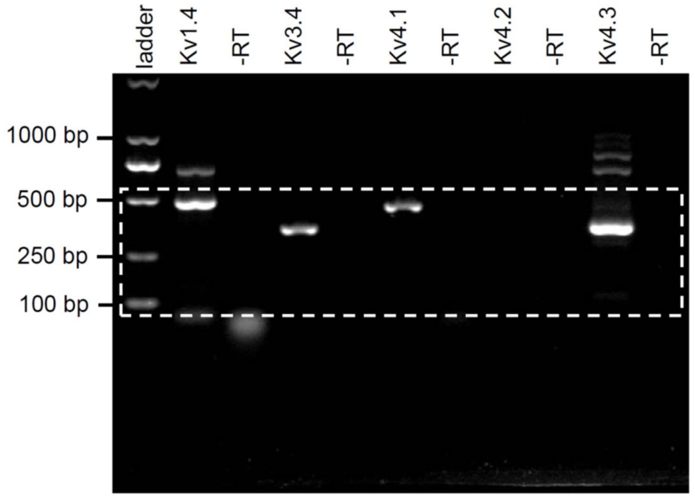

Figure S10: RT-PCR analysis of Kv1.4, Kv3.4, Kv4.1, Kv4.2, and Kv4.3 in the TGs of naïve mice. Shown is the expanded image of RT-PCR for Kv channels presented in Fig. 5A.

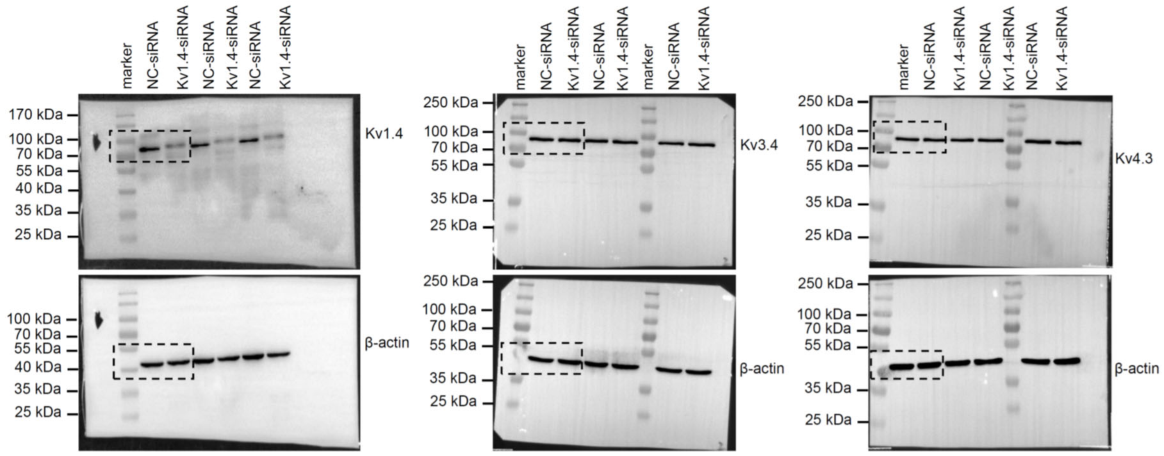

Figure S11: The protein expression of Kv1.4 was attenuated by intra-TG injection of Kv1.4-siRNA. Shown is the expanded image of Western blot for Kv1.4, Kv3.4, and Kv4.3 against a loading control, β-actin, presented in Fig. 5F. Blots are representative of three experiments.

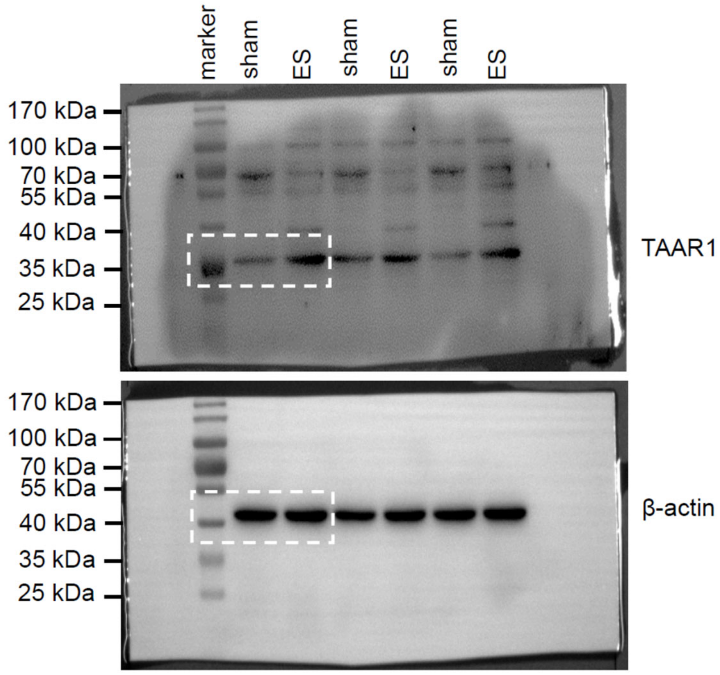

Fig. S12: The protein expression of TAAR1 in mouse TGs on day 5 after ES or sham surgery. Shown is the expanded image of Western blot for TAAR1 against a loading control, β-actin presented in Fig. 7F. Blots are representative of three experiments.

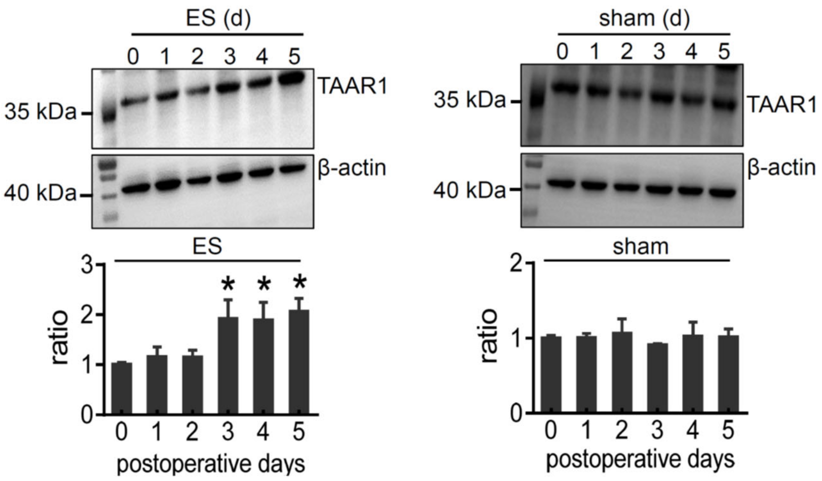

Fig. S13: Time course of TAAR1 protein expression in mouse TGs following sham or ES operation. *$p < 0.05$ *vs.* 0 day, one-way ANOVA. Blots are representative of three experiments.

Table S1: Validation of antibodies.

Antibodies for immunoblotting	
Rabbit anti-TAAR1 (Thermo Fisher Scientific, PA5-95704, 1:1000)	https://www.thermofisher.cn/cn/zh/antibody/product/TAAR1-Antibody-Polyclonal/PA5-95704
Rabbit anti-TAAR4 (Novus Biologicals, NBP3-10140, 1:1000)	https://www.novusbio.com/products/taar4-antibody_nbp3-10140
anti-Gαo (Cell Signaling Technology, #3975S, 1:1000,)	https://www.cellsignal.com/products/primary-antibodies/ga-o-antibody/3975?_=1681803908269&Ntt=3975&tahead=true
Rabbit anti-Gαi (Cell Signaling Technology, #5290S, 1:600,)	https://www.cellsignal.com/products/primary-antibodies/ga-i-antibody/5290?_=1681804003082&Ntt=5290&tahead=true
Rabbit anti-PKC$_{\theta}$ (Cell Signaling Technology, #13643, 1:500)	https://www.cellsignal.com/products/primary-antibodies/pkcq-e1i7y-rabbit-mab/13643
Rabbit anti-Kv1.4 (Thermo Fisher Scientific, PA5-85937, 1:800)	https://www.thermofisher.cn/cn/zh/antibody/product/KV1-4-KCNA4-Antibody-Polyclonal/PA5-85937
Rabbit anti-Kv4.3 (Thermo Fisher Scientific, PA5-95211, 1:600)	https://www.thermofisher.cn/cn/zh/antibody/product/KV4-3-KCND3-Antibody-Polyclonal/PA5-95211
Rabbit anti-Kv3.4 (Thermo Fisher Scientific, PA5-106236, 1:500)	https://www.thermofisher.cn/cn/zh/antibody/product/KV3-4-KCNC4-Antibody-Polyclonal/PA5-106236
Rabbit anti-β-actin antibody (Abcam, ab8227, 1:2000)	https://www.abcam.cn/products/primary-antibodies/beta-actin-antibody-ab8227.html
Goat Anti-Rabbit IgG H&L (HRP) (Abcam, ab6721, 1:5000)	https://www.abcam.cn/products/secondary-antibodies/goat-rabbit-igg-hl-hrp-ab6721.html
Antibodies for immunostaining	
Rabbit anti-TAAR1 (Thermo Fisher Scientific, PA5-115999, 1:300)	https://www.thermofisher.cn/cn/zh/antibody/product/TAAR1-Antibody-Polyclonal/PA5-115999
Mouse anti-NeuN (Cell Signaling Technology, #94403s, 1:600)	https://www.cellsignal.com/products/primary-antibodies/neun-e4m5p-mouse-mab/94403?_=1681803895350&Ntt=94403&tahead=true
Mouse anti-GS (Abcam,	https://www.abcam.cn/products/primary-

ab64613, 1:1000)	antibodies/glutamine-synthetase-antibody-3b6-bsa-and-azide-free-ab64613.html
Mouse anti-CGRP (Abcam, ab81887, 1:500)	https://www.abcam.cn/products/primary-antibodies/cgrp-antibody-4901-ab81887.html
Mouse anti-NF200 (Abcam, ab215903, 1:500)	https://www.abcam.cn/products/primary-antibodies/neurofilament-antibody-nf421--nfl736-ab215903.html
IB4-fluorescein isothiocyanate (Sigma–Aldrich, L2895, 5 μg/ml)	https://www.sigmaaldrich.cn/CN/zh/product/sigma/l2895
Alexa Fluor 555-conjugated goat anti-rabbit IgG (Cell Signaling Technology, #4413s, 1:300)	https://www.cellsignal.com/products/secondary-antibodies/anti-rabbit-igg-h-l-f-ab-2-fragment-alexa-fluor-555-conjugate/4413?_=1681804057362&Ntt=4413&tahead=true
DyLight 488-conjugated goat anti-mouse IgG (Cell Signaling Technology, #4408s, 1:300)	https://www.cellsignal.com/products/secondary-antibodies/anti-mouse-igg-h-l-f-ab-2-fragment-alexa-fluor-488-conjugate/4408?_=1681804155799&Ntt=4408&tahead=true

Table S2: Primer sequences for RT-PCR analysis

Gene		Primer sequences, 5’ to 3’	Amplicon size, bp
PKCη	sensee	GCTATCTGAGGGTCCGCATC	343 bp
	anti-sense	AGATCCACCCAGCCCTCGAA	
PKCθ	sensee	GCAGTTCTGCTCTTGCCCTA	370 bp
	anti-sense	GGCGACATCCTTACCCTTCC	
PKCδ	sensee	ACAGTCTATGCGTAGTGAGGAG	303 bp
	anti-sense	ATGTCGATGTTGAAGCGTTC	
PKCε	sensee	ATTCCTGCTTGCTTTGGTTT	424 bp
	anti-sense	GCTCTGGTGGTCTTTGTGCT	
Kv1.4	sensee	ACAAGTGATCGCTGCTGTCTA	485 bp
	anti-sense	GGTAGACGCAGTTCCAGCAG	
Kv3.4	sensee	GTGTCCGGAAAGAGACCTGC	344 bp
	anti-sense	GCACGTGCACTACACACAAG	
Kv4.1	sensee	CTGGGTGGTGAGATCATGGG	443 bp
	anti-sense	AGAGCACTAGCCACCCATCT	
Kv4.2	sensee	GAGCCTTTGTCACACTCCGA	399 bp
	anti-sense	CTGCTCGTTGGTTTTGGTGG	
Kv4.3	sensee	CTCCTCCGACTCTTTGCG	343 bp
	anti-sense	CAGGGATGCTTGTGAACTTGCT	
